# Microprojection arrays applied to skin generate mechanical stress, induce an inflammatory transcriptome and cell death, and improve vaccine-induced immune responses

**DOI:** 10.1038/s41541-019-0134-4

**Published:** 2019-10-11

**Authors:** Hwee-Ing Ng, Zewen K. Tuong, Germain J. P. Fernando, Alexandra C. I. Depelsenaire, Stefano C. Meliga, Ian H. Frazer, Mark A. F. Kendall

**Affiliations:** 1 Delivery of Drugs and Genes Group (D2G2), Australian Institute for Bioengineering and Nanotechnology, The University of Queensland, St Lucia, QLD 4072 Australia; 2 The University of Queensland Diamantina Institute, The University of Queensland Translational Research Institute, 37 Kent Street, Woolloongabba, QLD 4102 Australia; 3 Present Address: Vaxxas Pty Ltd, Translational Research Institute, 37 Kent Street, Woolloongabba, QLD 4102 Australia; 4 Present Address: MRC Laboratory of Molecular Biology, University of Cambridge, Cambridge, UK; 5 Present Address: The Australian National University, Canberra, ACT 2600 Australia

**Keywords:** Translational research, Adjuvants

## Abstract

Chemical adjuvants are typically used to improve immune responses induced by immunisation with protein antigens. Here we demonstrate an approach to enhance immune responses that does not require chemical adjuvants. We applied microprojection arrays to the skin, producing a range of controlled mechanical energy to invoke localised inflammation, while administering influenza split virus protein antigen. We used validated computational modelling methods to identify links between mechanical stress and energy generated within the skin strata and resultant cell death. We compared induced immune responses to those induced by needle-based intradermal antigen delivery and used a systems biology approach to examine the nature of the induced inflammatory response, and correlated this with markers of cell stress and death. Increasing the microprojection array application energy and the addition of QS-21 adjuvant were each associated with enhanced antibody response to delivered antigen and with induction of gene transcriptions associated with TNF and NF-κB signalling pathways. We concluded that microprojection intradermal antigen delivery inducing controlled local cell death could potentially replace chemical adjuvants to enhance the immune response to protein antigen.

## Vaccine delivery: Mechanical stress as an ‘adjuvant’

Adjuvants play an essential function in vaccine formulations by boosting immune responses to the delivered antigen. Mark A. F. Kendall and colleagues investigate the efficacy of vaccine delivered intradermally via Nanopatch^TM^—a 16 mm^2^ ultra-high-density array of 100 micron needles. Systems analysis comparisons demonstrate that depending on the force applied, the Nanopatch^TM^ triggers skin transcriptomic changes similar to that elicited by the adjuvant QS-21, including evidence of localised cell death and inflammatory gene expression. Flu vaccine delivered by Nanopatch^TM^ elicits anti-hemagglutinin titers equivalent to that observed with conventional intradermal syringe delivery of vaccine plus adjuvant. Sterile mechanical stress elicited by the application of microneedles might therefore be a viable replacement to the use of conventional needles.

## Introduction

Adjuvants were first deployed in vaccines in the 1930s and have since been included as a component of many vaccines, delivered by needles to enhance systemic antibody responses to immunising proteins.^1–3^ However, only a few chemical adjuvants are licensed for the use in human vaccines, including various Alum compounds, Monophosphoryl Lipid A and squalene-based MF59™. Adding adjuvant to a vaccine does not always improve the induced protective response^4^ and some currently available vaccines, such as Influenza and Tuberculosis, still induce levels of protection less than required to be broadly effective. Improved vaccines may come from better adjuvants and from deeper understanding of how adjuvants induce enhanced protection against infection.

In contrast to intramuscular immunisation, intradermal antigen delivery accesses the high density of antigen-presenting cells in the skin. Here we tested whether a vaccine delivery device targeting the skin could use the energy of application to induce localised physical inflammation that might circumvent the need for chemical adjuvants. The idea of applying energy to the skin to improve immune responses is not new.^5^ Examples of approaches that have been investigated include acoustic (e.g., sonophoresis^6,7^), electrical (e.g., electroporation^8,9^) and others (e.g., lasers^10,11^). Mechanical means of delivery of vaccines, including ballistic gene guns delivery of vaccines, have been explored since the 1990s, although the effects of energy of application has not been investigated.^12,13^ Making mechanical delivery devices suitable for practical vaccination programmes is challenging, as the design requirements, materials and energy sources are constrained by issues of costs and compactness.

Here we quantify how energy delivered to the skin generates local inflammation that leads to improved systemic immune responses. We compare the local inflammatory response to mechanically delivered energy with that produced by injection of a typical subunit vaccine, using a simple, practical medical device to deliver mechanical energy to the skin that is suitable for widespread vaccination of people.

To evaluate the use of this mechanical energy as an adjuvant, we used a prototype (Nanopatch™) medical device, an array comprising of ultra-high density (20 000 cm^−2^) silicon microprojections, 4 × 4 mm in dimension (Supplementary Fig. 1), to deliver antigen to the skin in mice. Each microprojection has a cylindrical-shaped body and a tapered conical tip, measuring about 100 µm in length. Precision, accuracy and repeatability of immunisation is achieved by dynamically applying the Nanopatch™ to the skin, using a spring-loaded applicator.^14^ We have previously shown that the Nanopatch™ enhances immune responses when contrasted with needle and syringe intradermal delivery of antigen.^15^ However, the mechanism of physical immune enhancement was not investigated. The most relevant literature is based on the adjuvanting effects of sterile inflammation and the release of danger-associated molecular patterns (DAMPs). We hypothesise that controlled mechanical impact during the application of the application of the Nanopatch™ would cause limited and localised cell death, and the resultant innate inflammatory response would contribute to enhanced vaccine-induced adaptive immune responses.^16^

In this study, we compared conventional intradermal administration of antigen with a needle and syringe to antigen delivery by Nanopatch™, a device engineered to use mechanical energy to penetrate the outermost layer of the skin, and evaluated the response to vaccine delivered into the skin. Using a systems vaccinology approach, we examined how the mechanical response induced by the Nanopatch™ is associated with gene and pathway transcription changes that may contribute to the improved antibody response.

## Results

### Determining the stress distribution and cell death invoked in the skin by the application of vaccine delivery devices

**Fig. 1:**
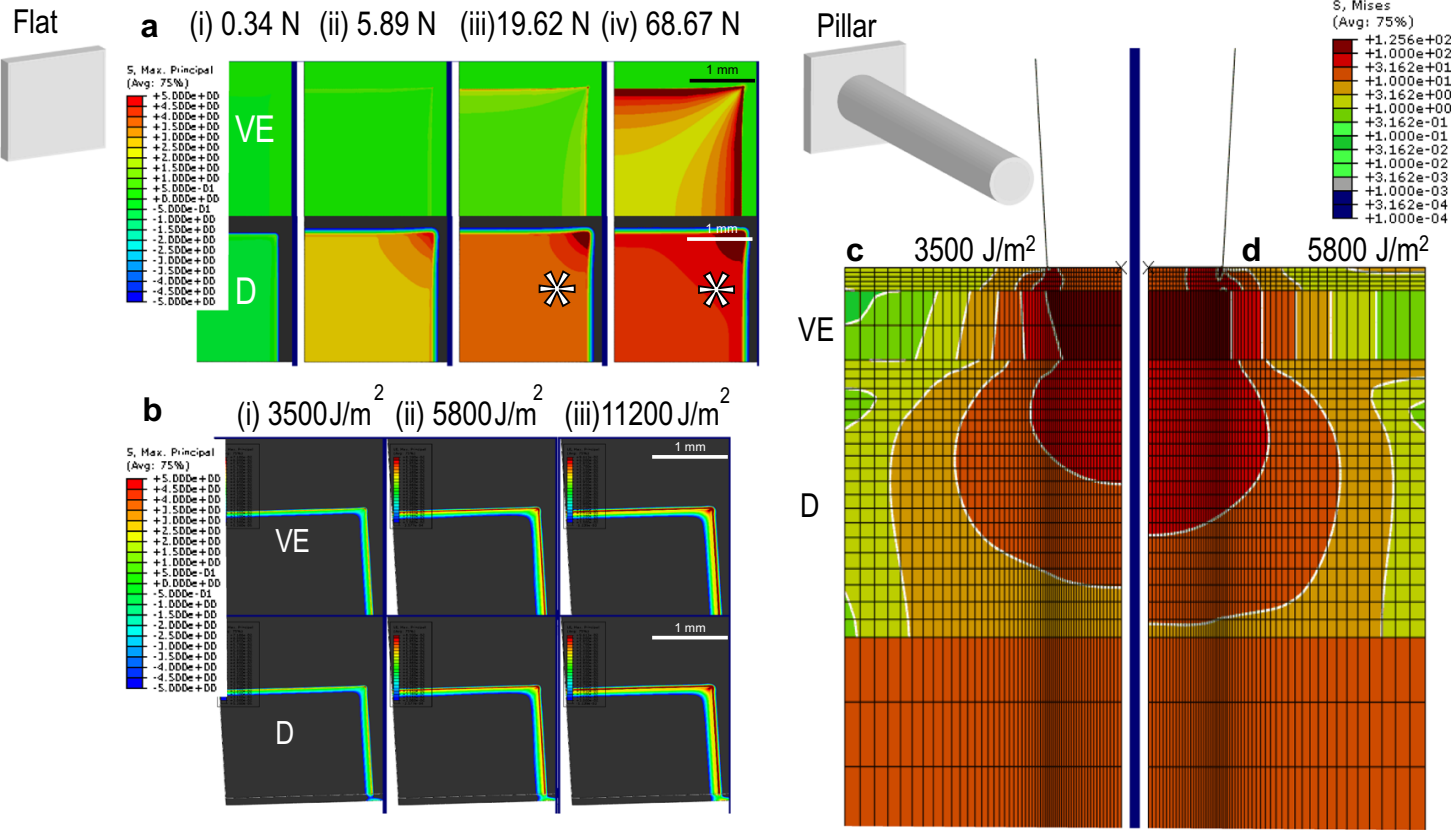
Mathematical modelling of stress in the skin (VE viable epidermis, D dermis) following application of a flat plate surface or a pillar array statically with a weight, or dynamically with a constant energy. **a** Flat plate surface applied with static weights of (i) 0.34 N, (ii) 5.89 N, (iii) 19.62 N or (iv) 68.67 N, showing distribution of maximum principal stress. **b** Flat plate array applied with dynamic energies of (i) 3500 J/m^2^, (ii) 5800 J/m^2^ or (iii) 11,200 J/m^2^, showing distribution of maximum principal stress. **c** Pillar array applied with dynamic energies of (i) 3500 J/m^2^ or (ii) 5800 J/m^2^, showing distribution of Von Mises stress. Red represents high stress and blue represents low or no stress. Limitation of model capturing small-scale stress response including edge stress marked with * in **a** demonstrated by cell death assay in Fig. 2

**Fig. 2:**
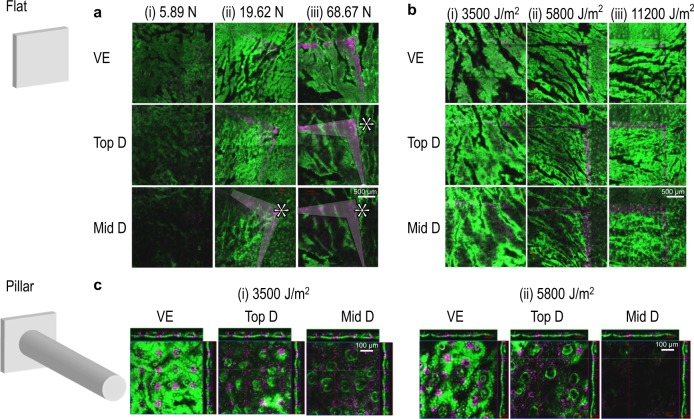
Cell death profile of skin (VE viable epidermis, D dermis) following application of a flat plate surface or a pillar array statically with a weight, or dynamically with a constant energy. **a** Flat plate array applied with static weights of (i) 5.89 N, (ii) 19.62 N or (iii) 68.67 N. **b** Flat plate array applied with dynamic energies of (i) 3500 J/m^2^, (ii) 5800 J/m^2^ or (iii) 11,200 J/m^2^. **c** Pillar microprojection array applied with dynamic energies of (i) 3500 J/m^2^, (ii) 5800 J/m^2^ or (iii) 11,200 J/m^2^. Green represents viable cells and magenta represents dead cells. Limitation of model capturing small-scale stress response including edge stress marked with * in Fig. 1a demonstrated by cell death assay in **a**

To examine how mechanical application of stress/energy to the skin is transmitted and how it is linked with skin cell death, we applied a skin deformation and fracture model^17^ to estimate the consequences of static or dynamic application of flat plate, pillar or cylindrical–conical microprojection array (Supplementary Figs 2 and 3). Using choices of energy and penetration data from previous work, we compared the spatial distribution of stress calculated by simulation with experimentally measured cell death in mouse ear skin. The modelled stress distribution of both the static and dynamic application of the flat plate (Fig. 1a, b) was qualitatively consistent with observed cell death (Fig. 2a, b). The corners and edges of the application area induced localised cell death in all skin layers, in agreement with the higher stress predicted by the model in these regions (1–5 MPa). However, in the dermis, experimentally measured distribution of cell death upon static application was discrepant with the numerical model. This is likely due to the difficulty of the model capturing small-scale stress response including edge stress when simulating large-scale mechanical interaction with a flat plate (Figs 1 and 2; viable epidermis (VE) and dermis marked with *). Dynamic application of a pillar array generated cell death tightly localised around the pillar heads in the VE, whereas the dead cell distribution expanded to cover most volume in the top and mid-dermis (Fig. 2c). This is consistent with the stress distributions calculated by the model (Fig. 1c, d), which shows stresses extending to a larger diameter in the dermis than in the VE.

Skin cell death was observed following static flat array loading above ~0.34 N (Fig. 2a) and for all dynamic impact conditions tested (>3500 J/m^2^). Comparing data from in silico modelling with observed cell death in ear tissue across the range of applied pressure and energy, we consistently observed that cell death was associated with local stresses above ~5 MPa (Figs 1 and 2).

**Fig. 3:**
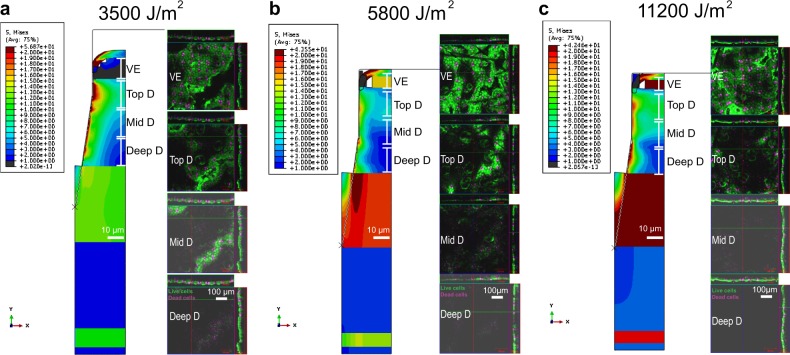
Von Mises stress model and cell death profile of a single microprojection when applied to the skin (VE viable epidermis, D dermis). Microprojections applied at **a** 3500 J/m^2^, **b** 5800 J/m^2^ and **c** 11,200 J/m^2^ into the different skin layers. Von Mises stress models represented by shades of red and blue colours (red: higher stress; blue: lower or no stress), and cell death profile represented by green and magenta colours (green: viable cells; magenta: dead cells)

The methods used to test skin cell mechanical tolerance for the flat plate and the pillar array were next used with a cylindrical–conical microprojection array, tested across a range of dynamic application energies (3500, 5800 and 11,200 J/m^2^; Supplementary Table 1). Cell death was closely localised around each microprojection in the VE, top- and mid-dermis (Fig. 3). This is in agreement with the calculated stress distribution, which peaked close to the microprojection tip-skin contact surface (Fig. 3). By comparing the co-localisation of stress magnitude along the stress contours with the observed cell death, we derived a stress threshold for cell death between 5 and 15 MPa, similar to the value deduced from the flat plate and pillar array with static and dynamic loading (>5 MPa). The average modelled and cell death imaging validated stresses dynamic application were 30 MPa in the VE and 3 MPa in the dermis with the inclusion of edge stresses in the 16 mm^2^ area.

### Adjuvant-like gene transcription changes following mechanical impact into the skin with or without antigen and adjuvant

Having assessed by theoretical modelling the stress distribution in the skin induced by mechanical stress, and compared this with imaged experimental cell death, we next use a systems approach to determine the transcription changes induced by mechanical stress to the skin. To do this, we use whole tissue RNA-sequencing (RNA-seq) to analyse changes in local gene expression, comparing untreated skin with intradermal antigen delivery using a cylindrical–conical microprojection array (a Nanopatch™ prototype) at 3500 and 11,200 J/m^2^, or needle and syringe antigen delivery, which delivers ~50 J/m^2^.

**Fig. 4:**
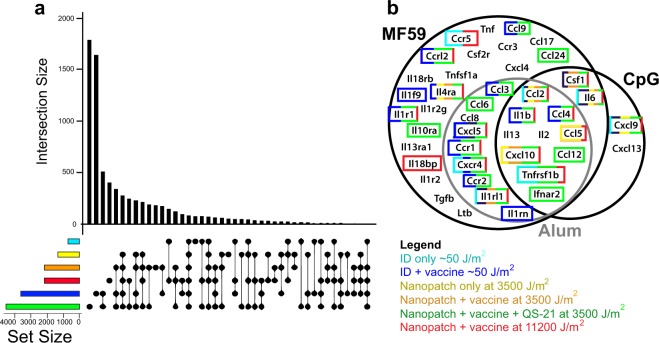
Differentially expressed genes (DEGs) in the skin following different modes of intradermal immunisation. **a** Total number of DEGs for each vaccination condition (horizontal bars colour coded as per legend) and distribution of number of DEGs (vertical black bars), found either commonly shown as connected black circles or exclusively shown as single black circle. **b** Comparative analysis assessing the regulation of cytokine and chemokine genes following physical and chemical adjuvantation (Mosca et al.^18^). Genes found to be significantly upregulated in both the Mosca et al.^18^ and gene set from this study are highlighted with colour-coded boxes as per legend. Different vaccination conditions are colour coded (turquoise: needle and syringe intradermal (ID) 50 J/m^2^ with saline; blue: ID 50 J/m^2^ with vaccine protein; yellow: Nanopatch™ 3500 J/m^2^ blank; orange: Nanopatch™ 3500 J/m^2^ with vaccine protein; green: Nanopatch™ 3500 J/m^2^ with vaccine protein and QS-21; red: Nanopatch™ 11,200 J/m^2^ with vaccine protein). All groups *n* = 3 except naive samples *n* = 6

Application of microprojection arrays coated with influenza vaccine and purified Quillaja saponin adjuvant (QS-21) induced the highest numbers of differentially expressed genes (DEGs; 4591 DEGs) shown in Fig. 4a. Application of influenza vaccine-coated microprojection array at 3500 J/m^2^ (2195 DEGs) or at 11,200 J/m^2^ with vaccine protein (2200 DEGs) induced more DEGs than application of a microprojection array without vaccine (1354 DEGs). Needle and syringe intradermal injection of saline induced the lowest numbers of DEGs (711 DEGs), whereas needle and syringe intradermal delivery of vaccine protein induced a higher number of DEGs (3670 DEGs). These results suggest that QS-21, influenza vaccine and the physical impact of a microprojection array could each contribute substantially to the induction of gene expression in the skin.

**Fig. 5:**
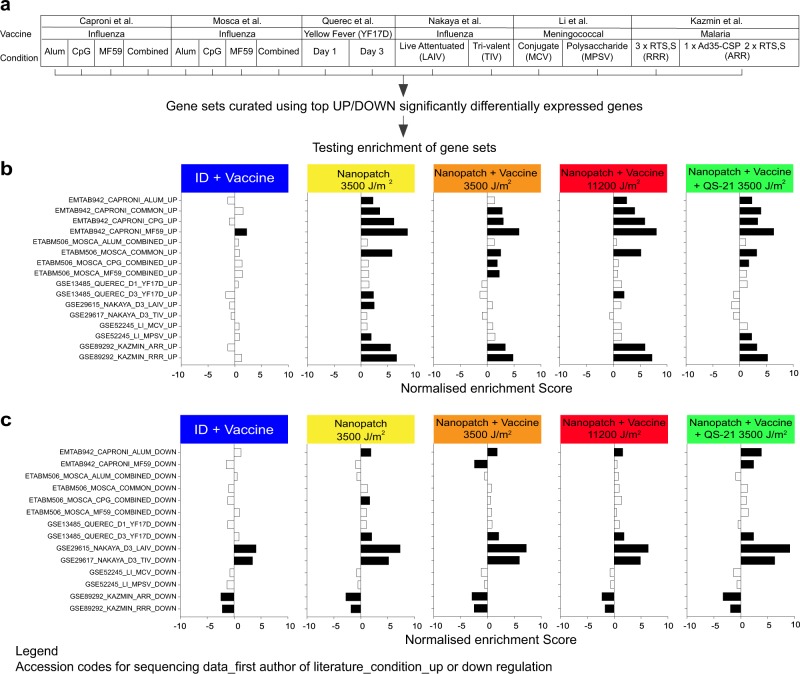
Gene set enrichment analysis (GSEA) of upregulated or downregulated gene sets curated from related vaccination mircoarray studies. **a** An illustration of related microarray studies (first row), with the vaccine (second row) and the corresponding condition/variable (third row). **b**, **c** Peak normalised gene set enrichment scores for the (*t* statistic) ranked gene lists for each group is displayed as a bar chart. Each bar chart corresponds to each vaccination condition, needle and syringe intradermal (ID) 50 J/m^2^ with vaccine protein, Nanopatch™ 3500 J/m^2^ blank, Nanopatch™ 3500 J/m^2^ with vaccine protein, Nanopatch™ 3500 J/m^2^ with vaccine protein and QS-21, and Nanopatch™ 11,200 J/m^2^ with vaccine protein compared with unimmunised control. The **b** upregulated and **c** downregulated gene sets are plotted against normalised enrichment score with significance represented by black horizontal bars (nominal *p* value < 0.05 and FDR *q*-value < 0.25). All groups *n* = 3, except naive samples *n* = 6

To contextualise our findings, we aligned our DEG data with those from a study by Mosca et al.,^18^ in which a ‘core adjuvant’ response gene signature was observed with their intramuscular administration of chemical adjuvants (Alum, CpG and MF59™) in comparison with phosphate-buffered saline (PBS) control. DEGs that were found to be shared between the Mosca et al.^18^ dataset and our dataset were highlighted, revealing many DEGs that are common to both datasets (Fig. 4b). We expanded this analysis to perform a systematic comparison between our RNA-seq DEG datasets and several vaccination studies with microarray data (Supplementary Table 2),^18–22^ using pre-ranked gene set enrichment analysis (prGSEA; Fig. 5a). Genes were ranked in our study by signed *t*-statistic values, to generate the ranked list of DEGs. Mouse genes sets were curated from Mosca et al.^18^ and Caproni et al.,^19^ whereas human genes sets were curated from Kazmin et al.,^22^ Li et al.^20^ and Nakaya et al.^23^ (Supplementary Table 3). Ranked DEG list following Nanopatch™ application without antigen at 3500 J/m^2^ displayed significant overlap with Mosca et al.^18^ gene signatures (IM, Alum/CpG/MF59 vs. PBS and CpG vs. PBS) and all 3500 and 11,200 J/m^2^ application groups displayed significant enrichment of the gene sets of Caproni et al.^19^ (IM, Alum/CpG/MF59 vs. PBS and Alum vs. PBS; Fig. 5b). Importantly, the upregulation of these genes was not observed following intradermal delivery of antigen by needle and syringe (Fig. 5b). Enrichment of the analogous human genes sets was also observed in Kazmin et al.,^22^ using malaria vaccine, and Li et al.,^20^ using the meningococcal polysaccharide quadrivalent vaccine (MPSV4/MPSV) in Fig. 5. However, there was little overlap between the downregulated gene sets in the various studies (Fig. 5c). Changes in gene expression in peripheral blood mononuclear cells following immunisation were not seen in our datasets. We provide a summary table for all the gene sets tested for the prGSEA in Supplementary Table 3. Overall, greater enrichment was generally seen with the 3500 and 11,200 J/m^2^ application groups than the needle and syringe intradermal 50 J/m^2^ energy application to the other microarray studies.

### Molecular pathway analysis of mechanical stresses and cell death revealed clustering of highly correlated genes associated with immunogenic roles

**Fig. 6:**
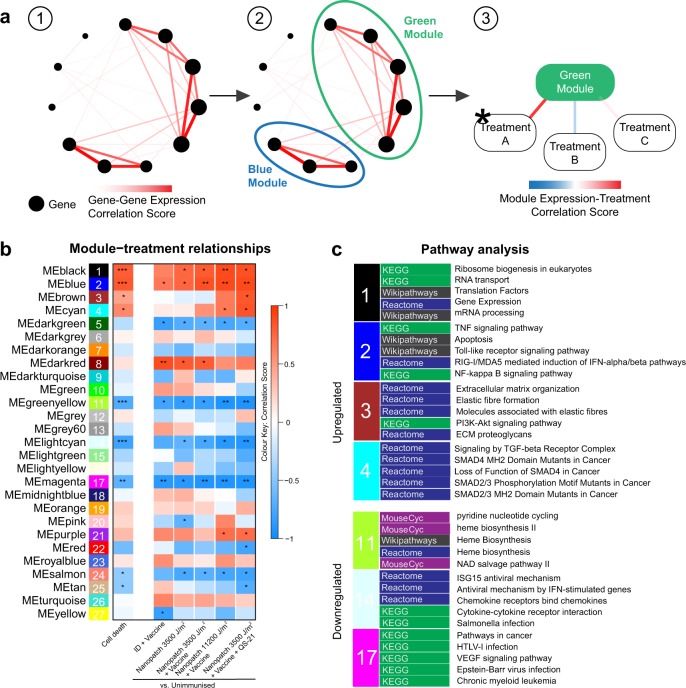
Weighted gene co-expression network analysis (WGCNA) of transcriptomic changes associated with intradermal antigen delivered by the needle and syringe (ID) or Nanopatch™ application. **a** An illustration of the analysis to identify gene changes of biological significance. (1) Strongly correlated (co-expressed) gene–gene pairs are calculated, (2) organised into modules and (3) tested for correlation with cell death assay results and treatment groups. **b** Module–treatment correlation score is presented as a gradient from blue to white, to red (red: positive correlation; white: no correlation; blue: negative correlation). Module–treatment correlation that attained statistical significance are denoted by *p ≤ 0.05, **p ≤ 0.001 and ***p ≤ 0.0001. **c** Pathway analysis was performed on modules that displayed significant correlation with the treatment groups. The top five KEGG, Reactome, MouseCyc or Wikipathways enriched by each gene module are shown. All groups *n* = 3, except naive samples *n* = 6

Next, to further investigate whether distinct gene networks were correlated with cell death or application energy, we applied a weighted gene co-expression network analysis (WGCNA; Fig. 6 and Supplementary Fig. 4). Figure 6b shows there are 27 distinct modules of highly correlated genes identified with WCGNA. Of these, seven gene modules in Fig. 6c were significantly positively correlated with cell death (Supplementary Table 4) or with application energy (Supplementary Table 1). These seven gene modules were further analysed. The blue module was upregulated significantly and correlated with increased cell death. This suggests a role for the genes in the blue module in immune enhancement from mechanical stimuli, as the difference was based on the energy applied during vaccination. Pathway enrichment using the Consensus Pathway Database (CPDB) showed that the top five enriched pathways in the blue module were tumour necrosis factor (TNF) signalling, apoptosis, Toll-like receptor (TLR) signalling, induction of interferon (IFN) α/β and nuclear factor (NF) κB signalling pathways. The top ten pathways of the seven significant gene modules of interest are shown in Supplementary Fig. 6 and all pathways for all colour modules are provided in Supplementary Data 1.

Complementary to the pathway analysis, ClueGO^24^ was used to visualise the most important/highly connected non-redundant biological functions enriched by the seven gene modules positively (black, blue, brown, cyan; Supplementary Fig. 7) and negatively (green–yellow, light cyan and magenta; Supplementary Fig. 8) correlated with cell death. The enriched pathways in these gene modules were similar to those demonstrated by CPDB analysis (Fig. 6c and Supplementary Fig. 8) and included pathways associated with immune signalling, cell death and antibody response. Pathways displaying module specificity (>60% genes from a single gene module) are highlighted. Several Gene Ontology (GO), Kyoto Encyclopaedia Genes and Genomes (KEGG) and Reactome pathways were consititued of genes found specifically in the blue or brown modules and not the black and cyan modules (Supplementary Fig. 7). The blue module showed specificity for TLR signalling, cytokines and chemokines (i.e., interleukin (IL) 6, IL-17 and TNF) signalling, T-cell receptor signalling and apoptosis, while the brown module displayed module specificity for microglial activation, antigen presentation, extracellular matrix pathways and B-cell-related antibody response (Supplementary Fig. 7). The brown module was significantly correlated to cell death measurement and the correlation was most prominent and statistically significant with 3500 J/m^2^ + vaccine + QS-21 vaccination, and with 11,200 J/m^2^ + vaccine (Fig. 6b). Pathways enriched by negatively correlated modules (green–yellow, light cyan and magenta; Supplementary Fig. 8) were also analysed, which showed that GO functions related to immune cell migration, cell death and stress response (Supplementary Fig. 7A), whereas there was limited enrichment of the canonical KEGG and Reactome pathways (Supplementary Figs 8B and 8C). However, we noted that the three negatively correlated gene modules were common to all comparisons (Fig. 6b), which could be a physiological function, and there were too few clusters for further analysis (Supplementary Fig. 8). Hence, we conclude that they do not reveal specific changes that may pertain to physical adjuvantation.

**Fig. 7:**
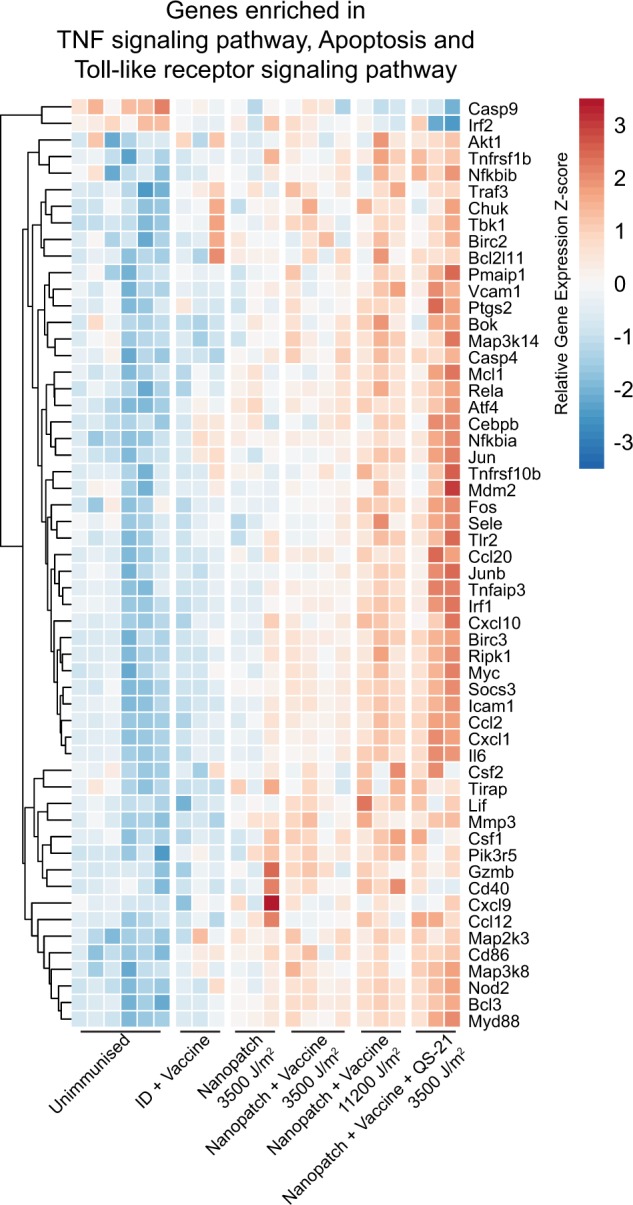
Relative expression heatmap of the genes enriched in TNF signalling, apoptosis and TLR-signalling pathway. Relative (scaled) expression of enriched genes in each sample is represented as a gradient from blue to white to red (red: high-expressing; *Z*-score = 3 and blue: low-expressing; *Z*-score = −3) and ordered according to their respective groups. All groups *n* = 3, except naive samples *n* = 6

Finally, genes enriched in the top three pathways in the blue module (Fig. 6c) were plotted into an expression heatmap in Fig. 7. Inspection of enriched genes from the immune-related pathways of the blue module showed a clear trend of gene expression, with the highest expression by Nanopatch™ vaccination with QS-21 (Fig. 7). Similar induction of genes was observed in Nanopatch™ vaccination at the highest application energy at 11,200 J/m^2^ and this trend of gene induction was observed to gradually decrease as the application energy reduces (Fig. 7). In contrast, the same genes in the needle and syringe intradermal vaccination groups at 50 J/m^2^ were closer to baseline levels presented in the unimmunised control (Fig. 7). Interestingly, without vaccine co-administered, Nanopatch™ at 3500 J/m^2^ enriched more of these genes than the needle and syringe intradermal 50 J/m^2^ with vaccine, which suggests that the vaccine is a poor inducer of physical immune enhancement and potentially induce immune response using different mechanisms.

### Relating delivery to the skin, with distinct levels of mechanical energy application, to invoke local differential expression in genes and resultant systemic antibody responses

**Fig. 8:**
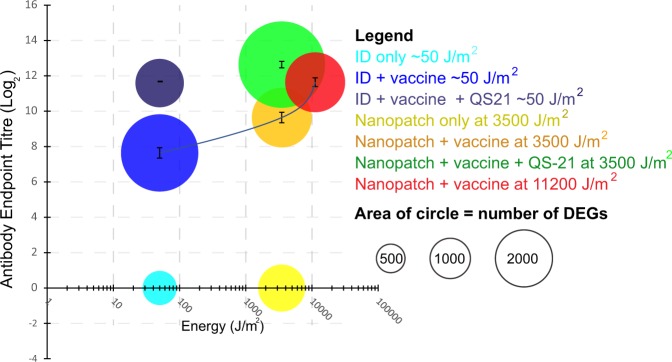
The average antibody endpoint titres (mean ± SEM), 21 days post intradermal immunisation, with the number of DEGs (area of circles) vs. various skin vaccination energies, 50, 3500 and 11,200 J/m^2^. ANOVA test was performed to HA-immunised samples (*p* < 0.001, refer to Supplementary Fig. 9, experiments for antibody responses were ran for three separate experiments with *n* = 4, 8 and 8)

The overarching requirement of vaccines is that they generate effective systemic immune responses. Therefore, we compared the antibody response to the immunisation strategies to assess whether the changes in local gene expression were correlated with the magnitude of the antibody response following immunisation with influenza vaccine. Hemagglutinin (HA)-induced antibody responses 21 days post immunisation were compared after intradermal administration by needle and syringe or by the Nanopatch™ at three application energies. With increasing application energy, the specific antibody responses increased significantly (one-way analysis of variance (ANOVA) *p* < 0.001; Fig. 8 and Supplementary Fig. 9). The antibody response to influenza vaccine when administered by the Nanopatch™ with QS-21 at 3500 J/m^2^ was similar to that following intradermal administration by the needle and syringe of vaccine with QS-21, and significantly higher than following administration by the Nanopatch™ at 3500 J/m^2^ but without QS-21 (one-way ANOVA *p* < 0.001; Supplementary Fig. 9). Increasing the application energy for the intradermal delivery device from 3500 to 11,200 J/m^2^ gave a similar increase in antibody response to the use of QS-21 (two-tailed *t*-test, *p* = 0.008).

Thus, delivering vaccine to the skin with increased application energy without chemical adjuvant induced similar improvements to antibody response to those achieved using a chemical adjuvant. This correlated with the pathway analysis (Fig. 6) and gene changes (Fig. 7) observed. The data support a hypothesis that enhancing physical adjuvantation by the use of a skin delivery device can increase the magnitude of a humoral immune response to a similar extent to those achieved by traditional chemical-based adjuvants.

## Discussion

This study demonstrated that a skin delivery device (Nanopatch™) applying mechanical energy could potentially replace the use of chemical adjuvant to improve vaccine-induced antibody response. Mathematical models simulating interaction of mechanical energy, applied by delivery devices to the skin resulting in spatial stress distribution in various skin layers, correlate consistently with cell death, with an estimated threshold of 30 MPa in the VE and 3 MPa in the dermis. Using a systems biology approach, investigation of the mechanism of vaccine-induced immune response associated with varying mechanical energies applied by the Nanopatch™ demonstrated the enrichment of immunologically relevant DEGs and pathways, and shown similar profiles to chemical adjuvants. Therefore, it is likely to be that the use of mechanical energy enhances immune response through the release of endogenous/native damage response molecules at the site of antigen delivery, creating local inflammation. Although this concept is not new, we have, in this study, quantified the relation between mechanical stimuli to the skin cell death, transcriptomic changes, and the resultant systemic antibody response.

Specifically, we correlated experimental cell death imaging with simulation of a theoretical skin deformation model in skin stress distribution of a vaccine delivery device. Macro- and micro-level analyses were performed using the whole array (Figs 1a, b and 2) and single projection (Figs 1c and 3). In all tested conditions, the stress-induced cell death was consistently observed at an average of 30 MPa in the VE and 3 MPa in the dermis (Figs 1 and 2). However, certain limitations apply. Calculated stress in the VE was likely affected by errors due to the large size of the finite elements used in modelling, and required to avoid element distortion and also to the interaction of skin model with the array base. Cell death in the deep dermis was not examined because of the likely effects on cell viability of split ear preparation. The agreement between the observed stress thresholds for cell death across the various models was otherwise consistent, even though skin viscoelasticity and size-dependent mechanical response cause an effective change in the elastic modulus from ~10^3^ Pa, when loaded statically by flat plate, to ~10^6^ Pa when loaded dynamically with the microprojection array. In particular, this ~1000-fold change in modulus causes an approximate directly proportional to ~1000-fold change of the stress magnitude generated by an equivalent deformation. This test conducted using dynamic microprojection thus effectively validates the stress-induced cell death model trained on static and dynamic flat plates and pillars, providing confidence that we can rationally engineer device designs and application conditions to achieve tailored cell death amounts and spatial distributions.

With the understanding of the interaction between the skin and mechanical energy, we reasoned that the magnitude of cell death should reflect the increased energy applied using the skin delivery device, which was observed to increase measured systemic immune response. This hypothesis was supported by the increased number of DEGs found in the site of vaccination (Figs 4a and 7). Transcriptomic profiling revealed an enhanced local immune response involving interactions in extracellular matrix, and triggering the IL-6, IL-17 and TNF signalling pathways and antigen presenting through TLR signalling to activate B-cell receptor signalling (Fig. 6 and Supplementary Figs 6 and 7). These changes correlated with improvements in antibody response with higher mechanical energy application of antigen and were comparable to those induced by chemical adjuvants (Fig. 8). Thus, skin delivery by Nanopatch™ induced localised cell death as a result of controlled micro-trauma caused by application and acted as a ‘physical immune enhancement’ to prime immune cells for antigen uptake and induced response.

The Nanopatch™ is designed to be a practical, needle-free way of delivering vaccine to the skin with adjustable mechanical energy for improved immune responses. However, there are other ways the energy has been transmitted for vaccine delivery. Previous studies have shown that laser illumination vaccination, at a dose of ~90 J/cm^2^ of application energy, demonstrated an improvement of antigen uptake and antigen-specific antibody response.^10,25–27^ This improvement possibly reflected application energy from a mixture of sources, including mechanical and thermal emission. A prior study from our group (Charkraborty et al.^28^) demonstrated that heat-induced sterile inflammation enhanced CD8^+^ T-cell responses. We have also demonstrated similar enhancement of CD8^+^ T-cell responses^27,29^ and antibody response^16,30^ in previous studies using 80- to 250-fold lower application energy than the laser illumination. An earlier delivery system, the ballistic gun uses mechanical energy to power DNA or protein antigens formulated in microparticles into the skin at high speed (200–800 m/s).^31^ Despite having a velocity of about 30 times higher than the Nanopatch™, systemic immune response were regularly equivalent to conventional delivery methods.^32,33^ Most of these technologies, and in particular microneedle/microprojection cutaneous delivery,^34–37^ have demonstrated equivalent or better responses to antigen than traditional needle and syringe. Gene gun and laser illumination deliver enhanced responses to antigen but require an additional complex entity to power the delivery (e.g., pressurised helium gas or motorised by electronics), potentially increasing the cost of vaccination and the size of the delivery device.

Thus, microprojection arrays (such as the Nanopatch™) may be a preferred mode of skin delivery of antigen, using simple application by mechanical means to target skin layers with high abundance of antigen-presenting cells. The improved immune response with higher mechanical application energy enhances TNF signalling, apoptosis, TLR signalling and induction of IFN-α/β and NF-ΚB signalling (Figs 6 and 7, and Supplementary Fig. 7). Activation of these pathways at the transcriptome level is likely a reflection of an overall enhanced localised response to antigen delivery.

We acknowledge that RNA transcript changes do not necessarily translate to changes at the protein level. Other studies have used post-vaccination sera for the detection of cytokines and chemokines to identify transcriptomic changes induced by their delivery systems, however it was largely restricted to selected immune related protein responses (e.g., IgG response, IL-6, IL-12, IL5 and CCL5).^18,29,38^ The published literature covers transcriptomic data from mice and humans, at different tissue sites including peripheral blood, skin and muscle biopsy, and has employed different analytic methods, including microarray or RNA-seq and at time points day 0, day 3 or day 7 post vaccination with one to three vaccine doses. Despite these analytic variables, comparison of the currently presented transcriptome data with the curated gene sets from publicly available vaccinology datasets highlighted similarities in the transcriptomic changes induced by physical immune enhancements to those induced by chemical adjuvants (i.e., Alum, CpG and MF59). The mechanical impact applied by the Nanopatch™ is thus likely to induce similar biological effects to chemical adjuvants^18,19^ and should result in the induction of an equivalent or better vaccination response.

Future studies of physical immune enhancements in humans are warranted. Due to genetic dissimilarity and skin differences, the translation of physical immune enhancers in a different species might change. Even though there are homologous genes in mice and human, the mechanical properties of human skin require further understanding, e.g., skin thickness,^39^ viscoelasticity^40^ or diseases in the skin can potentially affect the interaction between the skin and delivery devices; hence, this changes the systemic immune response invoked. Further understanding of stress-induced cell death in the skin should advance the rational design of next-generation vaccine and skin delivery technology. In summary, our findings support adoption of mechanical delivery systems such as the Nanopatch™ as a safe and effective vaccination approach that capitalises on the use of controlled mechanical energy to stress the skin cells and induce native/endogenous physical immune enhancements from the skin as a mode for enhancing vaccine-induced immunogenicity.

The microneedle technology has attracted considerable interest as a vaccine delivery and the ability to mitigate risks associated with conventional vaccination method with the needle and syringe. Two recent human clinical trials by Rouphael et al.^41^ and Fernando et al.^42^ demonstrated the potential of the microneedle technology as a vaccine delivery system. Both studies demonstrated well-tolerated reactions on the skin and at least an equivalent immune response compared with the conventional vaccination method. Furthermore, Rouphael et al.^41^ has shown that the microneedle technology can be self-administered, which could potentially remove the need of healthcare workers during vaccination.

However, these microneedles are slightly different to the ones used in this study (mouse prototype), in terms of projection density, length, morphology, array dimensions, application speed and material of the patch (dissolving vs. silicon), this affects the interaction between microprojection tip-skin contact (i.e., skin stress distribution, skin fracture/cell death threshold, vaccine dissolution in the skin, immune regulation from mechanical stress, to name a few). To add on to the complexity, preclinical studies in naive animals showed superior immunogenicity and vaccine efficacy post patch vaccination, whereas human volunteers are likely to have pre-existing immunity from previous exposure, making it harder to measure the improvement due to the application of a microneedle/patch.

Although there are challenges to overcome, the logistical and administration aspect of the vaccination using microneedle/patch are attractive. Within the next few years, microneedle technology is likely to play a vital role in vaccine delivery technology and could replace the needle and syringe.

## Methods

### Animals

All methods performed in this study were carried out in accordance with National Health and Medical Research Council (NHMRC) guidelines and were approved by The University of Queensland Animal Ethics Committee. All animal care and experiments were conducted in accordance with NHMRC (Australia) guidelines and with the approval of The University of Queensland animal ethics committee under AIBN/556/12/ARC/NHMRC/SMART or AIBN/043/16/ARC/NHMRC/SMART/WHO. Female C57BL/6J mice of 6–8 weeks were used per condition and they were maintained under specific pathogen-free condition with food ad libitum in the animal facility of the Australian Institute of Bioengineering and Nanotechnology, The University of Queensland.

### Vaccine formulation

Intanza®2013 (Sanofi Pasteur, Lyon, France) is a trivalent influenza subunit protein vaccine containing 90 µg/ml HA proteins from each influenza strains, A/California/7/2009 (H1N1) pdm09-like strain, A/Victoria/361/2011 (H3N2)-like strain and B/Wisconsin/1/2010-like strain. The formulations were calculated to deliver 30 ng of Intanza®2013, with or without 1.5 µg of QS-21 (Desert King International, San Diego, CA, USA) to each of the mouse model based on Supplementary Fig. 1, with Dulbecco’s PBS (dPBS; Gibco/Life Technologies) as the diluent.

### Transcutaneous immunisation

ID injections were prepared by diluting Intanza ®2013 in dPBS to the appropriate dose (30 ng) and administered to the ventral side of murine right ears.

To induce physical immune enhancements while vaccinating into the skin, the Nanopatch™ was used. These Nanopatch™ were manufactured as described elsewhere,^43^ measuring 4 × 4 mm with a density of 20,000 microprojections per cm^2^ and microprojections were 110 µm long. The Nanopatch™ were cleaned with 70% ethanol, rinsed with MilliQ water and air dried. The Nanopatch™ vaccine formulation consisted of 1% methylcellulose (Methocel 60 HG; Fluka/Sigma-Aldrich), Intanza®2013 and dPBS as the diluent. Vaccine formulation was dry coated on to the Nanopatch™ by a 70° and 20° angle nitrogen gas jet as described previously.^44^ One patch per mouse was used to vaccinate mice with a spring-loaded applicator at a constant velocity as specified in Supplementary Table 1, to penetrate microprojections into the ear skin of mice, left for 2 min to allow the reconstitution and diffusion of the dry-coated vaccine. The amount of vaccine delivered into the skin was quantified using a radioactive tracer and calculated as delivery efficiency (Supplementary Fig. 1). Vaccinations using the Nanopatch™ were calculated accordingly and no radioactive tracer was included.

### Mathematical modelling of mechanical induction of stress on to the skin

Briefly, static application allows the array (with mass of 0.035, 0.6, 2 or 7 kg, inducing 0.34, 5.89, 19.62 and 68.67 N, respectively) to rest on the skin for 2 min.

Dynamic application requires an applicator to fire the array at a constant energy (3500, 5800 or 11,200 J/m^2^) and the arrays are rested on the skin for 2 min, similar to the transcutaneous immunisation. The stress distributions were computed using the finite element model of the skin developed in Meliga et al.^17^ and were solved using Abaqus (Abaqus 6.11; Dassault Systemes Simulia, Corp., Providence, RI, USA). Specifically, the mouse ear was represented as a seven-layer material constituted by a 70 μm-thick cartilage layer sandwiched between the ventral and dorsal skin tissues having the following geometry: a 5 μm-thick stratum corneum (SC), a 15 μm-thick VE and a 60 μm-thick Dermis (D). The viscoelastic skin behaviour was simulated using the Ogden model; hence, hyperelastic parameters varied with the skin-probe contact area and deformation velocity according to Crichton et al.^45^ measurements. For example, the 11,200 J/m^2^ microprojection application was simulated using respectively the following Young’s moduli and stretch exponents: 187 MPa and 5.77 for the SC, 2.7 MPa and 162 for the VE, 401 MPa and 15.5 for the D, 0.02 MPa and 115 for the Cartilage. The complete set of rate and size-dependent moduli and exponents is summarised in Meliga et al.^17^ Similar to that in our previous work,^17^ we used the following properties for SC, VE, D and Cartilage: mass densities were 1.3, 1.1, 1.27 and 1.3 g cm^−3^, respectively. Poisson’s ratio was set to 0.45. For the microprojection simulations, penetration was simulated using a ductile failure model with critical stresses at the onset of fracture of 70, 2 and 12 pJ μm^2^, and damage dissipation energies of 35, 1 and 6 pJ/µm^2^ for the SC, VE and D, respectively. The probes were assumed to be rigid and have a friction coefficient of 0.4.

### Sample collection

Ear skin samples were obtained 4 h post vaccination and was snapped frozen using liquid nitrogen. All biopsies were weighted and kept at −80 °C for further RNA-seq analysis.

Blood was collected by retro-orbital bleeds on day 21. Blood was kept at room temperature for 2 h for clotting before centrifugation at 10,000 × *g* for 5 min to separate sera from whole blood. Sera were kept at −80 °C for further serological analysis.

### Cell viability staining using multi-photon microscopy

Skin samples were prepared and cellular viability was assessed similar to a previous study,^15^ using ×10 and ×20 air objectives (Zeiss, Germany). Briefly, the ear skin was split, cartilage was carefully removed and stained using a mixture of acridine orange (0.03 mg/ml) and ethidium bromide (0.1 mg/ml), labelling live (green) and dead (magenta) cells, respectively. Positive controls were pre-treated with ice-cold methanol before staining. Multi-photon microscopy (MPM) images were taken from four to six ear skin sample from different mouse per condition, except for naive condition (*n* = 2; from different mouse). MPM images taken (one ×10 overview image, one ×20 edge image and one ×20 centre image, unless otherwise specified) using software LSM510 and image acquisition using ZEN (both from Zeiss, Germany). A representative image was used.

### Serological analysis—antigen-specific antibody response using enzyme-linked immunosorbent assay

ntigen-specific antibody (day 21 sera) was measured by enzyme-linked immunosorbent assay (ELISA) similar to previous study.^16^ Briefly, 3 µg/ml of antigen (Intanza 2013) was diluted and used to coat ELISA plates (Nunc Maxisorp, ThermoFisher) overnight at 4 °C. The plates were blocked with 0.4% bovine serum albumin (BSA) in PBS and were used to determine the antigen-specific antibody titres. Sera were diluted to 1:100 with 0.4% BSA in PBS, then serially diluted and incubated for 2 h in room temperature. Washing of plates were done five times using 0.02% Phosphate-Buffered Saline, Tween-20 0.05% (PBST) and secondary antibody; anti-mouse IgG HRP (G-21040, Invitrogen/ThermoFisher) were diluted 1:1000 with 0.4% BSA in PBS to obtain a final concentration of 1 µg/ml and this was added and incubated for 1.5 h. Colour development was performed using ABTS (2,29-azino-bis3-ethylbenzthiazoline-6-sulfonic acid; A-1888, Sigma-Aldrich) as the substrate and measured at absorbance of 405 and 490 nm. Endpoint titres were calculated as described elsewhere.^46^ According to Frey et al.,^46^ endpoint is defined as the reciprocal of the highest dilution of a serum that gives a reading above the cutoff. This cutoff is established by the control sera from mock immunisation or unimmunised (true negative controls), ran at similar serial dilution of the same ELISA plate, and expressed as the SD multiplied by a factor based on the number of negative controls.

### Statistical analysis

Statistical analyses were performed using GraphPad Prism version 7.02 for Windows (GraphPad Software, La Jolla, CA, USA, www.graphpad.com). All data represented were expressed as the mean ± SEM, for trend observations. The ANOVA was performed for multiple group comparison and two-tailed unpaired Student’s *t*-test was performed as appropriate with single comparison. A difference was considered statistically significant when *p* < 0.05.

### RNA extraction, purification and sequencing

Ear skin samples were collected and stored at −80 °C until being processed. TissueRuptor® (9001274; Qiagen) was used to disrupt and homogenise skin in 1 ml of QIAzol Lysis Reagent. Total RNA were extracted using Qiagen RNeasy® Microarray Tissue Kit (73304; Qiagen) with an additional genomic DNA removal step (RNase-free DNAse set; 79254; Qiagen) according to manufacturer’s protocol. Purity and quality of RNA were validated by Nanodrop 1000 (ThermoScientific) and 2100 Bioanalyser (Agilent Technologies). RNA samples were stored at −80 °C until being processed. Complementary DNA libraries were made using TruSeq RNA Sample Preparation kit (RS122-2001/2; Illumina) and sequencing was run as a 50 bp single-end lane with at least 10 million read per sample on Illumina HiSeq 2000 instrument performed by Australia Genome Research facility. The sequencing data described in this study are deposited in ArrayExpress accession E-MTAB-7482.

### Sequence alignment, gene clustering and differential gene expression analysis

Sequences were processed using Galaxy server, Genome Virtual Lab.^47^ Quality control of raw sequence data was determined using the Phred Score obtained from Fast QC package. Reads were above a Phred Score of 25. Read alignment and transcript quantification was performed using Salmon.^48^ DESeq2 was used for differential gene expression analysis. Genes considered significantly regulated when *p* < 0.05.

### Gene set enrichment analysis

Pre-ranked GSEA was performed following a standard procedure.^49^ The gene signatures of Mosca et al. (E-TABM-506),^18^ Caproni et al. (E-MATB-942),^19^ Li et al. (GSE52245),^20^ Nakaya et al. (GSE29614 and GSE29615-GSE29617)^23^ and Qurec et al. (GSE 13485)^50^ were curated from publicly available microarray data. The top ~200 up or downregulated genes in each condition (vs. unimmunised, unless otherwise specified) were identified by differential gene expression using GEOquery and Limma R packages from the Bioconductor project. Mice Entrez Gene IDs were mapped to Human Entrez Gene IDs for analysis, using the HGNC Comparison of Orthology Predictions (downloaded 07/09/2017). However, the comparison with Pearton et al.^21^ could not be performed due to the lack of microarray data.

### Weighed gene co-expression network analysis

Regularised log-normalised expression data from RNA-seq experiments were generated using the DESeq2 and batch correction was performed using the ComBat package embedded within the surrogate variable analysis package from the Bioconductor R project.^51^ A weighted gene network was generated from the data by following the standard procedure of the WGCNA package.^52^ WGCNA provides a mean of identifying correlation patterns of genes across microarray or RNA-seq samples and subsequently finding modules of highly correlated genes/nodes via a hierarchical clustering approach coupled with topology overlap matrix-based dissimilarity measure. WGCNA constructs networks using a scale-free topology criterion where connectivity of genes/nodes follows a power law distribution where strongly connected gene–gene pairs are highlighted, whereas weakly connected pairs are penalised. This was found to be useful for the identification of intrinsic biologically meaningful modules of co-ordinately expressed genes.^53^ Briefly, the similarity (concordance) between gene expression profiles across the samples was measured using Pearson’s correlations. The co-expression similarities are converted into connection strengths and used to define the distance (dissimilarity) between genes (or nodes). The WGCNA package uses the topological overlap dissimilarity measure, as it was found to result in biologically meaningful modules.^53^ Genes with coherent expression profiles were grouped into modules using average linkage hierarchical clustering coupled with the topological overlap dissimilarity measure. The gene modules correspond to the branches of the resulting dendrogram. To identify modules that are significantly associated with external traits, the summary profile (eigengene) of each module was correlated with predefined external traits and looked for the most significant associations. According to the WGCNA package, the module eigengene is the representative value for a module and is defined as the first principal component of a module; the normalised linear combination of genes with the maximum possible variability in a module. To facilitate biological interpretation, pathway overrepresentation analysis for each gene module was performed using ConsensusPathDB (http://cpdb.molgen.mpg.de/MCPDB).^54^ The top five processes from KEGG and GO biological processes were reported. Top ten pathways hits from KEGG were plotted onto GraphPad Prism.

### Heatmap

Log2 normalised gene expression values for the respective genes were extracted from the expression matrix used for WGCNA analysis. The expression data were centred and scaled to a *z*-scale, which represents the expression values as SDs from the centre. The data were then converted to a colour scale from blue to white, to red, where blue indicates low expression and red indicates high expression. The heatmap was generated using the pheatmap R package.

### Reporting summary

Further information on research design is available in the Nature Research Reporting Summary linked to this article.

## Supplementary information

Supplementary text and figures

Reporting Summary

## Data Availability

Sequencing data that support the findings of this study have been deposited in ArrayExpress with accession code E-MTAB-7482.
