# Supplementary material for: Microprojection arrays applied to skin generate mechanical stress, induce an inflammatory transcriptome and cell death, and improve vaccine-induced immune responses
Source: NPJ Vaccines. 2019 Oct 11;4:41. doi: 10.1038/s41541-019-0134-4 (PMC6789026; doi:10.1038/s41541-019-0134-4)
Supplement: Supplementary file 1 — Supplementary text and figures [file 41541_2019_134_MOESM1_ESM.pdf]

# Microprojection arrays applied to skin generate mechanical stress, induce an inflammatory transcriptome and cell death, and improve vaccine-induced immune responses

Authors: Hwee-Ing Ng[1,2‡*], Zewen K Tuong[3,4‡], Germain JP Fernando[1,2], Alexandra CI Depelsenaire[1,2], Stefano C Meliga[1], Ian H Frazer[3*], Mark AF Kendall[1,5]

## Supplementary Information

### Supplementary Figures

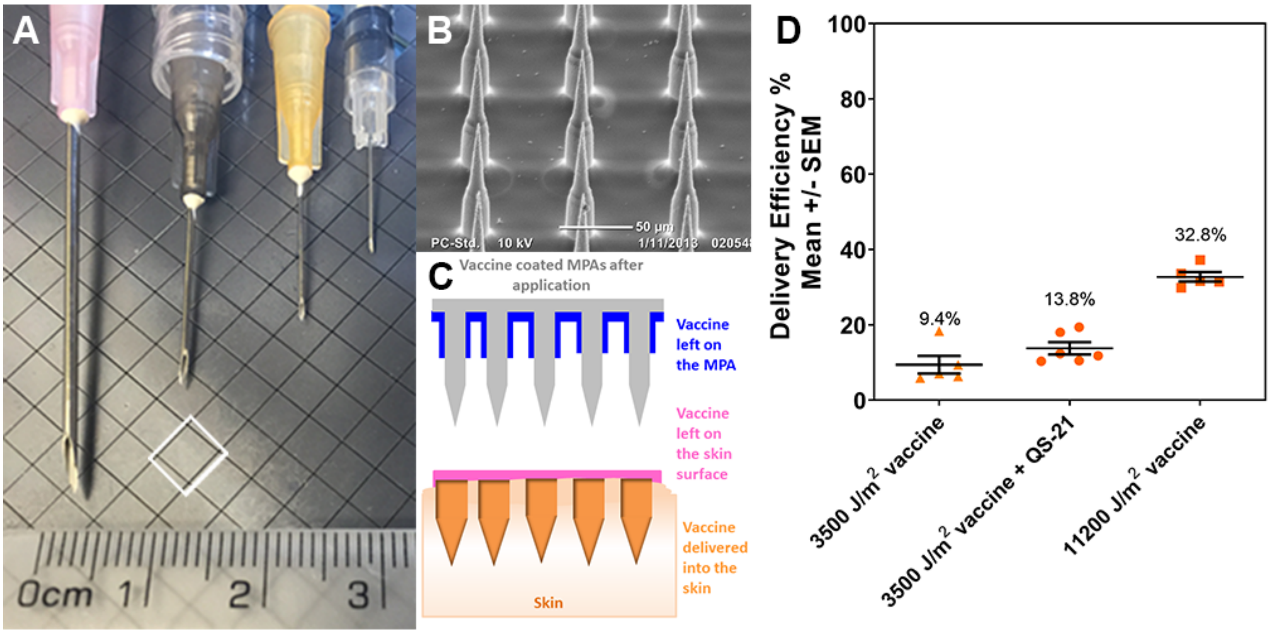


**Supplementary Figure 1**: A) A comparative photo of the different needle gauges (18, 23, 25 and 31 gauges) with the background of Nanopatch™, the white square is highlighting one 4 by 4 $mm^2$ Nanopatch™. B) A scanning electron microscope image of the Nanopatch™ in the background. C) An illustration of a coated Nanopatch™ applied into skin and where the vaccine will localise after application. D) The delivery efficiency of the patch is the percentage of the vaccine that was delivered into the skin. The quantification of delivered vaccine was done as previously described[1, 2].

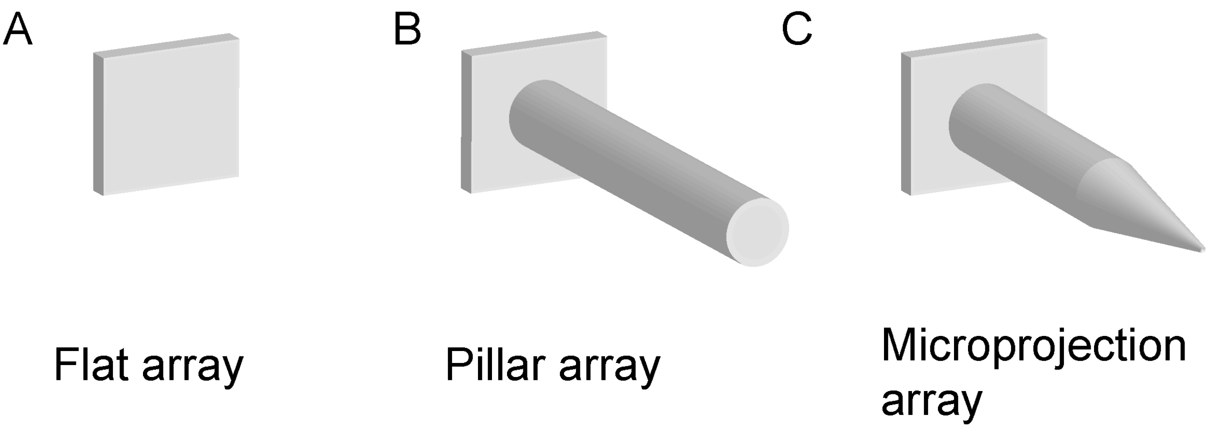


**Supplementary Figure 2**: An illustration of the various arrays, A) flat, B) pillar and C) microprojection, used for mathematical modelling.

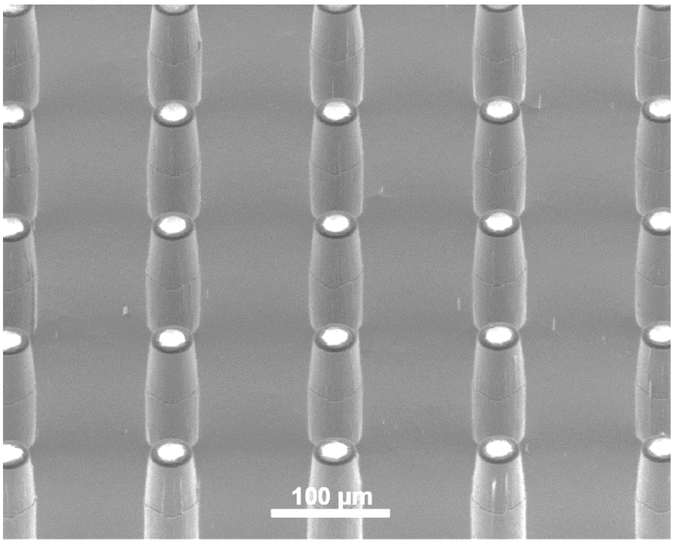


**Supplementary Figure 3**: A scanning electron microscopy (SEM) of pillar array shown in Supplementary Figure 2.

## External traits input for weighed gene co-expression network analysis (WGCNA) from previous published studies

Our previous studies with cell death caused by the Nanopatch™ has been extensively studied and published before. Depelsenaire et al[3] demonstrated the difference of cell death in comparison with intradermal (ID) injection showing the correlation between the increased antibody responses with the increased cell death. While Ng et al[4] used different patch vaccine formulation, with the inclusion of chemical adjuvant (QS-21) and demonstrated the increased cell death upon the addition of QS-21. In this study, we extracted information from

the two studies for our weighed gene co-expression network analysis (WGCNA; Figure 6A) to show correlation of cell death (Supplementary Table 4) to differentially expressed genes (DEGs) demonstrated in this study.

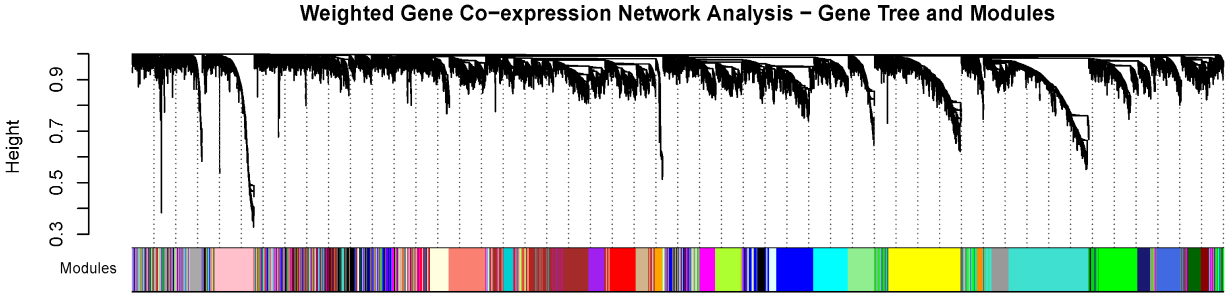


**Supplementary Figure 4:** The gene-gene dissimilarity dendrogram and assigned gene modules colour from WGCNA. Refer to Figure 6 for more information.

## Cell death analysis between 3500 J/m$^2$ and 1120 J/m$^2$ application energy to skin

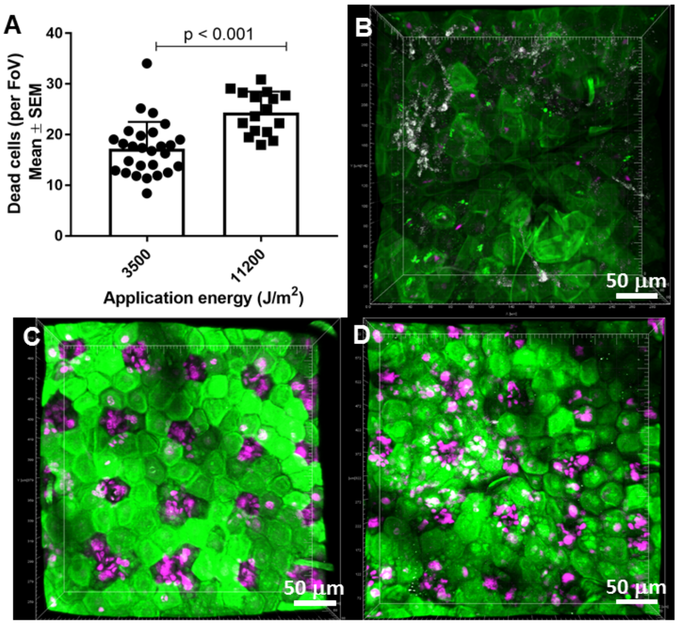


**Supplementary Figure 5**: Comparison of cell death (Mean%) for datasets Nanopatch 3500 J/m$^2$ vaccine and Nanopatch 11200 J/m$^2$ + vaccine. A) Dead cell percentage from both 3500 J/m$^2$ and 11200 J/m$^2$, and multi-photon microscopy image from B) a naive skin, C) post 3500 J/m$^2$ application skin and D) post 11200 J/m$^2$ application skin (n=4 or 5; 4 images per sample, 2 images from the array edge and 2 image from the centre of the array).

## Pathways analysis using CPDB from significant gene modules

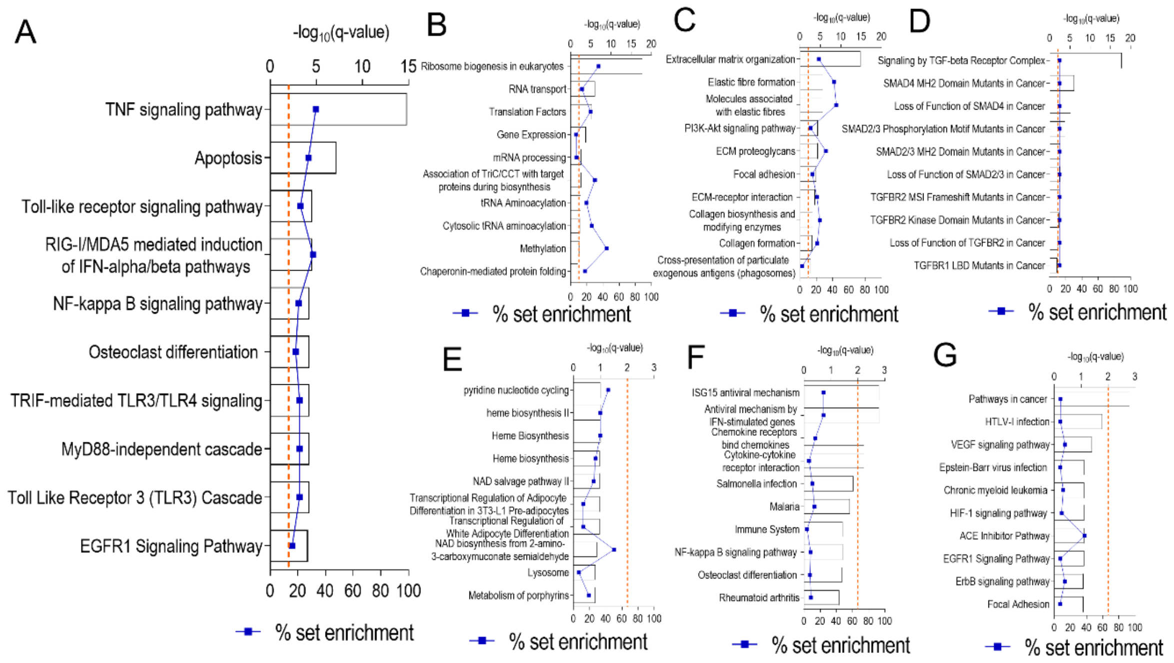


**Supplementary Figure 6**: Consensus pathway database (CPBD) pathway analysis from extracted gene data from the 6 modules of WGCNA (Figure 6), gene sets from A) blue, B) black, C) brown, D) cyan, E) greenyellow, F) lightcyan and G) magenta modules demonstrates the percentage of set enrichment and its respective q-value.

## ClueGO analysis of enriched gene ontology networks by all the significant correlated modules from WGCNA

Using the data obtained from WGCNA (Figure 6 and Supplementary Figure 3), genes enriched from each of the colour modules were analysed. Each of the nodes (circles) are highlighted in their respective module colours, where more than 50% of the genes are enriched. This will visualise overlapping pathways that contains co-enriched genes obtained from existing databases.

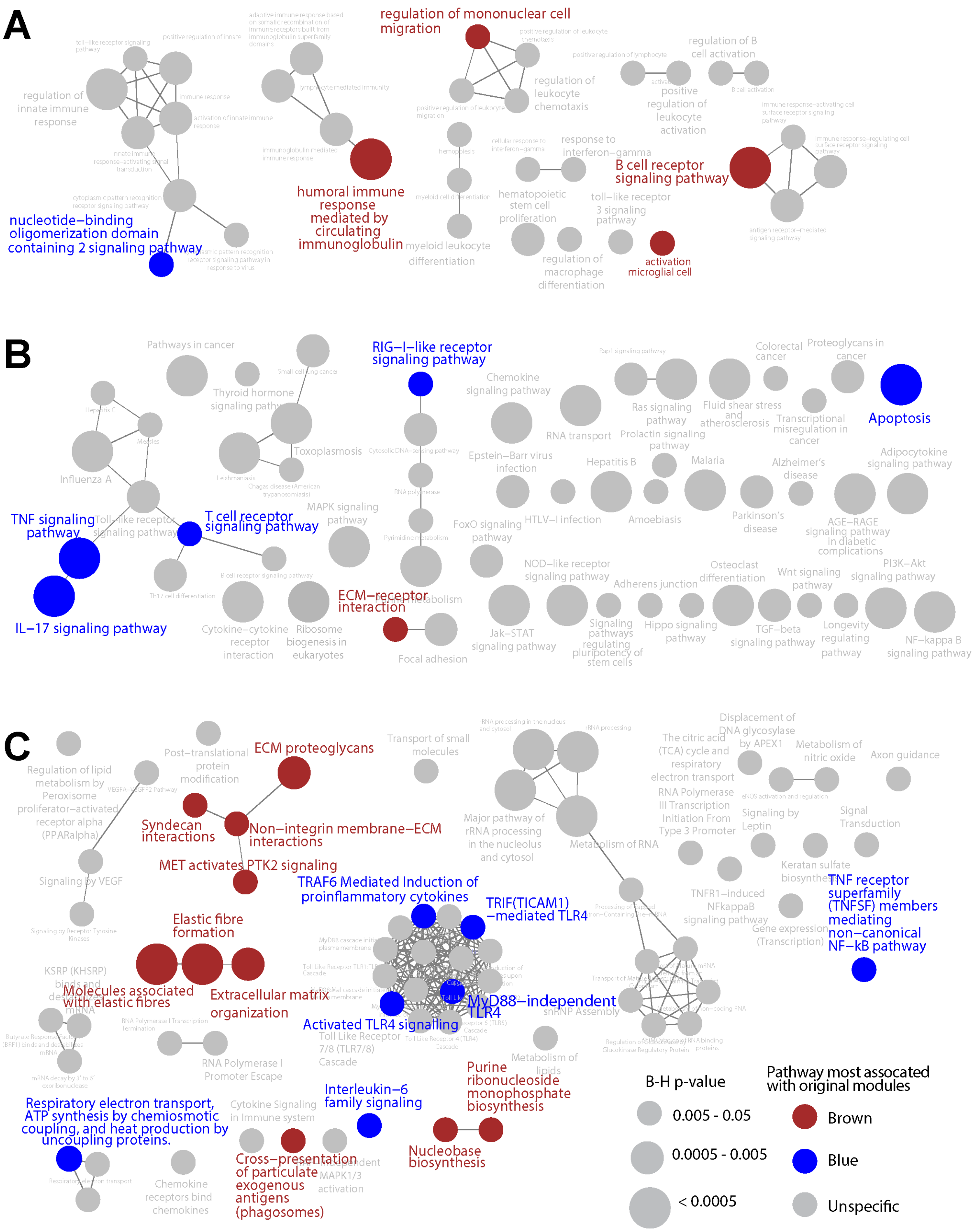


**Supplementary Figure 7**: ClueGO analysis of enriched gene ontology networks by all the significant positively correlated modules from WGCNA (black, blue, brown and cyan) for A) Gene Otology Biological Processes (GO), B) Kyoto Encyclopaedia of Genomes and Genes (KEGG), and C) Reactome databases. Coloured nodes indicate >50% of genes were contributed by either blue or brown modules; cyan and black modules do not have nodes

with >50% genes. Grey nodes indicate non-specific contribution. Size of nodes indicates the statistical significance of the enrichment analysis.

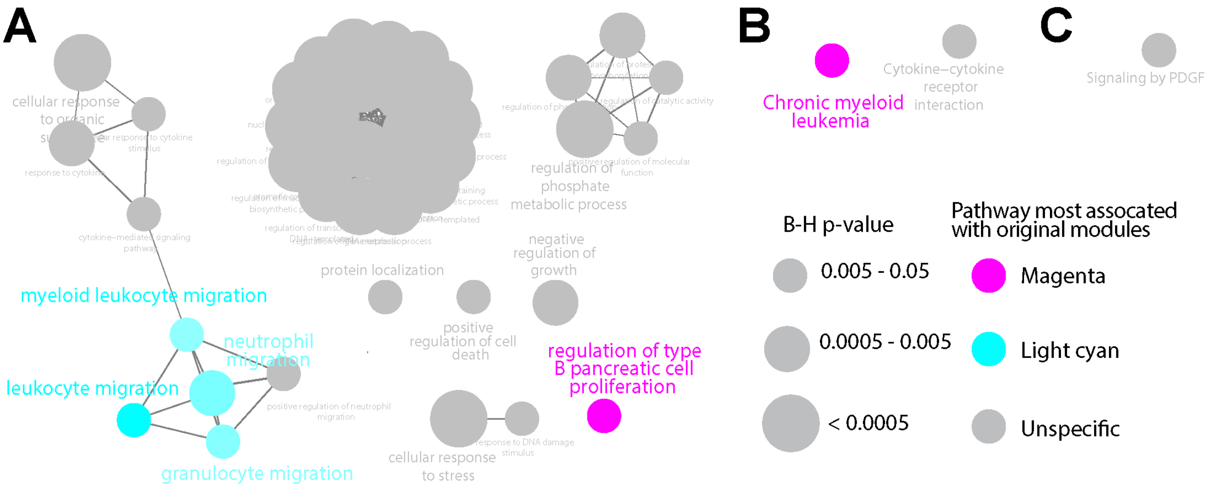


**Supplementary Figure 8**: ClueGO analysis of enriched gene ontology networks by all the significant negatively correlated modules from WGCNA (greenyellow, magenta and lightcyan) for A) Gene Otology Biological Processes (GO), B) Kyoto Encyclopaedia of Genomes and Genes (KEGG), and C) Reactome databases. Coloured nodes indicate >50% of genes were contributed by either lightcyan or magenta modules; greenyellow module does not have nodes with >50% genes. Grey nodes indicate non-specific contribution. Size of nodes indicates the statistical significance of the enrichment analysis.

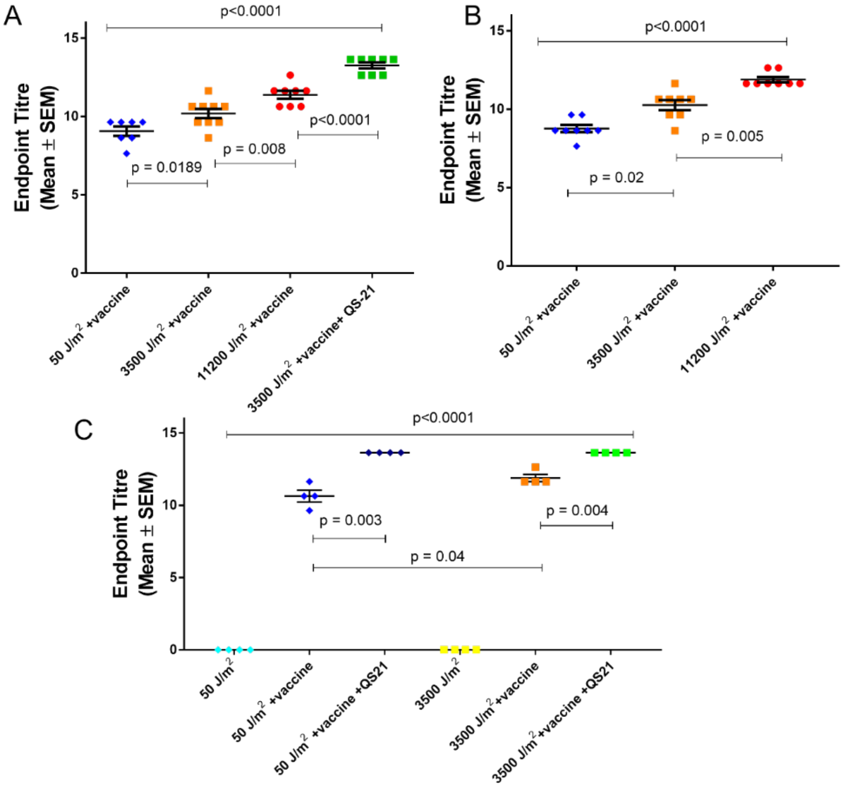


**Supplementary Figure 9:** Antibody endpoint titres, plotted as an average in Figure 8, with statistical comparison, n<4 per experiment, one-way ANOVA to compare more than two groups (top p value) and unpaired t-test was performed to compare between two groups (bottom p value), demonstrating significant differences between each of the application energy.

## Supplementary Table

**Supplementary Table 1**: Calculation of energy applied dynamically to the skin with a Nanopatch™. Spring loaded applicator weighs 0.35 kg and the calculation of the energy transferred is based on the weight of the applicator and the velocity of the Nanopatch, after triggering the applicator and before contacting the skin.

| **Application energy of dynamic application** | | | |
|---|---|---|---|
| Area | 16 $mm^2$ | | |
| Mass | 0.035 kg | | |
| Velocity | 1.8 m/s | 2.3 m/s | 3.2 m/s |
| Energy | 0.0567 J or 3500 $J/m^2$ | 0.0926 J or 5800 $J/m^2$ | 0.1792 J or 11200 $J/m^2$ |

**Supplementary Table 2**: The summary of related microarray studies used for GSEA comparison.

| Sample | Timepoint | Vaccine | Used as a comparison in this study | Reference |
|---|---|---|---|---|
| **Muscle biopsy** | 6 hours post vaccination | Influenza subunit antigen + adjuvant (Alum or MF59) | Yes | Caproni et al (E-MATB-942)[5] |
| **Muscle biopsy** | 6 hours post vaccination | Influenza subunit antigen + adjuvant (Alum, CpG, MF59 or a combination of CpG and MF59) | Yes | Mosca et al (E-TABM-506)[6] |
| **PBMCs** | Common regulation from multiple timepoints | Malaria vaccine (RTS,S) three doses (RRR) or adjuvanted With two doses(ARR) | Yes | Kazmin et al (RRR and ARR)[7] |
| **PBMCs** | 3 days post vaccination | Influenza (subunit antigen or live attenuated) | No common DEGs found | Nakaya et al (GSE29614 and GSE29615-GSE29617)[8] |
| **PBMCs** | 1 or 3 days post vaccination | Influenza (subunit antigen or live attenuated), meningococcal vaccine (polysaccharide or conjugate) or yellow fever virus vaccine | No common DEGs found | Li et al (GSE52245) [9] |
| **PMBCs** | 1 or 3 days post vaccination | yellow fever virus vaccine | Yes | Qurec et al (GSE 13485)[10] |
| **Skin explant culture** | 24 hours post vaccination | Influenza virus-like particle vaccine | No microarray found with publication | Pearton et al[11] |

## GSEA comparison to other chemical adjuvant studies without any similarities

Chemical adjuvant published studies were included in this study to compare the similarities to physical immune enhancers. The elegant studies by Li et al (GSE52245)[9] and Nakaya et al (GSE29614 and GSE29615-GSE29617)[8] demonstrated several differences compared to our studies; hence no similarities could be drawn from the comparisons. Specifically, Li et al (GSE52245)[9] used commonly regulated DEGs of PBMCs from human volunteers three days post meningococcal (MCV and MPSV4) vaccination while Nakaya et al (GSE29614)[8] used

PBMCs from human volunteers three days post influenza vaccine (TIV season 2007/08) vaccination and (GSE29615-GSE29617)[8] commonly regulated DEGs from human PMBCs comparing different types of influenza vaccines (TIV and LAIV vaccination season 2008/09) day three post vaccination, compared to their respective controls.

Several reasons that could explain the differences observed. Firstly, the direct localised genetic response at the site of vaccination were studied whereas PBMCs indirectly encounter the vaccine, after antigen processing, when entering the circulation, hence displaying tissue specific gene expression in agreement with others[12, 13]. Secondly, the sampling time point was four hours post vaccination for this study, while others sampled the PBMCs three days post vaccination. This allowed antigen presentation process to progress so as to observe gene expression changes from the vaccination site into the circulation (PBMCs). Thirdly, our animal models were C57BL/6 mice whereas in GSE52245, GSE29614 and GSE29615-GSE29617, human volunteers were tested. Despite that tissue specificity could be an issue; more similarities could be found in homologous tissues from different species than between diverse tissues of the same species in other study[13]. There could be contributions from the different vaccine that was used as model antigen in GSE52245, with MCV and MPSV[9].

## Significant concordance from Gene Set Enrichment Analysis (GSEA) leading edge comparison of various vaccination conditions to relevant publication

The different vaccination datasets (3500 J/m$^2$ + blank, 3500 J/m$^2$ + vaccine, 11200 J/m$^2$ + vaccine, 3500 J/m$^2$ +vaccine + QS-21 and ID 50 J/m$^2$ + vaccine) were compared to relevant vaccination studies using GSEA (Figure 5A).

**Supplementary Table 3:** GSEA result summary of the comparison between groups in this study (Nanopatch 3500 J/m$^2$ only, Nanopatch 3500 J/m$^2$ + vaccine, Nanopatch 11200 J/m$^2$ + vaccine, Nanopatch 3500 J/m$^2$ +vaccine + QS-21 and ID 50 J/m$^2$ + vaccine) with relevant vaccination studies, reflected on Figure 3.

| | **Vaccination gene sets** | **Size** | **Enrichment Score** | **Normalized Enrichment Score** | **Nominal P-value** | **FDR q-value** | **Rank at Max** |
|---|---|---|---|---|---|---|---|
| Nanopatch 3500 J/m$^2$ | GSE89292_KAZMIN_ARR_DOWN | 346 | -0.1311 | -2.8151 | <0.0001 | 0.0001 | 4318 |
| | GSE89292_KAZMIN_RRR_DOWN | 345 | -0.0871 | -1.8738 | 0.0107 | 0.0329 | 3243 |
| | EMTAB942_CAPRONI_MF59_DOWN | 187 | -0.0636 | -1.0029 | 0.4284 | 0.8672 | 5587 |
| | GSE52245_LI_MCV_DOWN | 53 | -0.0961 | -0.8238 | 0.6847 | 1.0000 | 8344 |
| | ETABM506_MOSCA_ALUM_COMBINED_DOWN | 103 | -0.0644 | -0.7649 | 0.7681 | 0.9159 | 9177 |
| | GSE52245_LI_MPSV_DOWN | 91 | -0.0522 | -0.5786 | 0.9565 | 0.9581 | 14675 |
| | EMTAB942_CAPRONI_MF59_UP | 272 | 0.4536 | 8.7320 | <0.0001 | <0.0001 | 3485 |
| | GSE29615_NAKAYA_D3_LAIV_DOWN | 348 | 0.3434 | 7.3562 | <0.0001 | <0.0001 | 6319 |
| | GSE89292_KAZMIN_RRR_UP | 335 | 0.3176 | 6.6956 | <0.0001 | <0.0001 | 4770 |
| | EMTAB942_CAPRONI_CPG_UP | 155 | 0.4283 | 6.1940 | <0.0001 | <0.0001 | 2554 |
| | ETABM506_MOSCA_COMMON_UP | 123 | 0.4502 | 5.8304 | <0.0001 | <0.0001 | 4025 |
| | GSE89292_KAZMIN_ARR_UP | 332 | 0.2660 | 5.5630 | <0.0001 | <0.0001 | 6237 |
| | GSE29617_NAKAYA_D3_TIV_DOWN | 231 | 0.2959 | 5.2223 | <0.0001 | <0.0001 | 6319 |
| | EMTAB942_CAPRONI_COMMON_UP | 31 | 0.5415 | 3.5721 | <0.0001 | <0.0001 | 3986 |
| | GSE29615_NAKAYA_D3_LAIV_UP | 300 | 0.1256 | 2.5106 | 0.0002 | 0.0005 | 6879 |
| | GSE13485_QUEREC_D3_YF17D_UP | 113 | 0.1922 | 2.3877 | <0.0001 | 0.0010 | 3988 |
| | EMTAB942_CAPRONI_ALUM_UP | 87 | 0.2089 | 2.2861 | 0.0008 | 0.0019 | 6258 |
| | GSE13485_QUEREC_D3_YF17D_DOWN | 24 | 0.3569 | 2.0890 | 0.0044 | 0.0061 | 4581 |
| | GSE52245_LI_MPSV_UP | 136 | 0.1447 | 1.9638 | 0.0057 | 0.0120 | 4575 |
| | EMTAB942_CAPRONI_ALUM_DOWN | 212 | 0.1139 | 1.9254 | 0.0076 | 0.0139 | 5886 |
| | ETABM506_MOSCA_CPG_COMBINED_DOWN | 28 | 0.2699 | 1.6967 | 0.0233 | 0.0441 | 5151 |
| | GSE13485_QUEREC_D1_YF17D_UP | 145 | 0.1089 | 1.5257 | 0.0591 | 0.0925 | 7099 |
| | ETABM506_MOSCA_MF59_COMBINED_UP | 263 | 0.0797 | 1.4945 | 0.0721 | 0.1007 | 11698 |
| | ETABM506_MOSCA_CPG_COMBINED_UP | 82 | 0.1332 | 1.4193 | 0.0999 | 0.1324 | 12093 |
| | ETABM506_MOSCA_COMMON_DOWN | 10 | 0.3398 | 1.3026 | 0.1588 | 0.1998 | 11265 |
| | ETABM506_MOSCA_ALUM_COMBINED_UP | 210 | 0.0748 | 1.2552 | 0.1877 | 0.2273 | 7350 |
| | GSE52245_LI_MCV_UP | 36 | 0.1657 | 1.1827 | 0.2437 | 0.2797 | 2864 |
| | ETABM506_MOSCA_MF59_COMBINED_DOWN | 24 | 0.1915 | 1.1264 | 0.3050 | 0.3230 | 13085 |
| | GSE29617_NAKAYA_D3_TIV_UP | 173 | 0.0730 | 1.1196 | 0.3000 | 0.3156 | 3105 |
| | GSE13485_QUEREC_D1_YF17D_DOWN | 40 | 0.1436 | 1.0741 | 0.3504 | 0.3503 | 4805 |

| | Vaccination gene sets | Size | Enrichment Score | Normalized Enrichment Score | Nominal P-value | FDR q-value | Rank at Max |
|---|---|---|---|---|---|---|---|
| Nanopatch 3500 J/$m^2$ + vaccine | GSE89292_KAZMIN_ARR_DOWN | 346 | -0.1353 | -2.9185 | <0.0001 | <0.0001 | 5184 |
| | GSE89292_KAZMIN_RRR_DOWN | 345 | -0.1177 | -2.5106 | <0.0001 | 0.0006 | 3770 |
| | EMTAB942_CAPRONI_MF59_DOWN | 187 | -0.1556 | -2.4658 | 0.0002 | 0.0007 | 8597 |
| | GSE13485_QUEREC_D3_YF17D_UP | 113 | -0.1128 | -1.3976 | 0.1095 | 0.2403 | 9562 |
| | GSE52245_LI_MCV_DOWN | 53 | -0.1389 | -1.1894 | 0.2431 | 0.4287 | 5363 |
| | GSE13485_QUEREC_D1_YF17D_UP | 145 | -0.0723 | -1.0118 | 0.4199 | 0.6345 | 11132 |
| | GSE29617_NAKAYA_D3_TIV_UP | 173 | -0.0648 | -0.9881 | 0.4606 | 0.5829 | 9162 |
| | GSE52245_LI_MPSV_DOWN | 91 | -0.0542 | -0.6093 | 0.9379 | 1.0000 | 14640 |
| | ETABM506_MOSCA_ALUM_COMBINED_DOWN | 103 | -0.0496 | -0.5886 | 0.9500 | 0.9522 | 1313 |
| | GSE29615_NAKAYA_D3_LAIV_DOWN | 348 | 0.3368 | 7.2624 | <0.0001 | <0.0001 | 5890 |
| | EMTAB942_CAPRONI_MF59_UP | 272 | 0.3127 | 5.9893 | <0.0001 | <0.0001 | 6980 |
| | GSE29617_NAKAYA_D3_TIV_DOWN | 231 | 0.3366 | 5.9825 | <0.0001 | <0.0001 | 5560 |
| | GSE89292_KAZMIN_RRR_UP | 335 | 0.2301 | 4.8563 | <0.0001 | <0.0001 | 5724 |
| | GSE89292_KAZMIN_ARR_UP | 332 | 0.1632 | 3.4274 | <0.0001 | <0.0001 | 4823 |
| | EMTAB942_CAPRONI_CPG_UP | 155 | 0.2097 | 3.0518 | <0.0001 | 0.0000 | 5148 |
| | EMTAB942_CAPRONI_COMMON_UP | 31 | 0.4278 | 2.8454 | <0.0001 | 0.0001 | 3720 |
| | ETABM506_MOSCA_COMMON_UP | 123 | 0.1997 | 2.5716 | 0.0004 | 0.0003 | 3690 |
| | ETABM506_MOSCA_MF59_COMBINED_UP | 263 | 0.1241 | 2.3112 | 0.0006 | 0.0017 | 9006 |
| | GSE13485_QUEREC_D3_YF17D_DOWN | 24 | 0.3642 | 2.1355 | 0.0022 | 0.0051 | 3746 |
| | ETABM506_MOSCA_CPG_COMBINED_UP | 82 | 0.1823 | 1.9423 | 0.0070 | 0.0144 | 10634 |
| | EMTAB942_CAPRONI_ALUM_DOWN | 212 | 0.1099 | 1.8649 | 0.0120 | 0.0202 | 4665 |
| | GSE52245_LI_MPSV_UP | 136 | 0.1107 | 1.5105 | 0.0629 | 0.1064 | 3268 |
| | EMTAB942_CAPRONI_ALUM_UP | 87 | 0.1292 | 1.4113 | 0.0987 | 0.1511 | 748 |
| | ETABM506_MOSCA_ALUM_COMBINED_UP | 210 | 0.0791 | 1.3338 | 0.1445 | 0.1938 | 8170 |
| | GSE52245_LI_MCV_UP | 36 | 0.1493 | 1.0684 | 0.3598 | 0.4687 | 1249 |
| | GSE29615_NAKAYA_D3_LAIV_UP | 300 | 0.0528 | 1.0512 | 0.3741 | 0.4652 | 4687 |
| | GSE13485_QUEREC_D1_YF17D_DOWN | 40 | 0.1187 | 0.8915 | 0.5866 | 0.6810 | 4802 |
| | ETABM506_MOSCA_MF59_COMBINED_DOWN | 24 | 0.1384 | 0.8142 | 0.7015 | 0.7684 | 14700 |
| | ETABM506_MOSCA_COMMON_DOWN | 10 | 0.2059 | 0.7937 | 0.7276 | 0.7606 | 13548 |
| | ETABM506_MOSCA_CPG_COMBINED_DOWN | 28 | 0.1013 | 0.6376 | 0.9071 | 0.9139 | 5585 |

| | Vaccination gene sets | Size | Enrichment Score | Normalized Enrichment Score | Nominal P-value | FDR q-value | Rank at Max |
|---|---|---|---|---|---|---|---|
| Nanopatch 11200 J/m$^2$ + vaccine | GSE89292_KAZMIN_ARR_DOWN | 346 | -0.1085 | -2.3068 | 0.0008 | 0.0037 | 8197 |
| | GSE89292_KAZMIN_RRR_DOWN | 345 | -0.0817 | -1.7353 | 0.0244 | 0.0566 | 8871 |
| | GSE52245_LI_MCV_DOWN | 53 | -0.0925 | -0.7952 | 0.7290 | 1.0000 | 2291 |
| | GSE29617_NAKAYA_D3_TIV_UP | 173 | -0.0472 | -0.7192 | 0.8281 | 1.0000 | 5416 |
| | GSE52245_LI_MPSV_DOWN | 91 | -0.0624 | -0.6960 | 0.8591 | 0.8512 | 12815 |
| | EMTAB942_CAPRONI_MF59_UP | 272 | 0.4238 | 8.0696 | <0.0001 | <0.0001 | 5742 |
| | GSE89292_KAZMIN_RRR_UP | 335 | 0.3443 | 7.2570 | <0.0001 | <0.0001 | 6818 |
| | GSE29615_NAKAYA_D3_LAIV_DOWN | 348 | 0.2963 | 6.3878 | <0.0001 | <0.0001 | 5342 |
| | GSE89292_KAZMIN_ARR_UP | 332 | 0.2835 | 5.9413 | <0.0001 | <0.0001 | 6818 |
| | EMTAB942_CAPRONI_CPG_UP | 155 | 0.4072 | 5.9113 | <0.0001 | <0.0001 | 4892 |
| | ETABM506_MOSCA_COMMON_UP | 123 | 0.3990 | 5.1791 | <0.0001 | <0.0001 | 5447 |
| | GSE29617_NAKAYA_D3_TIV_DOWN | 231 | 0.2800 | 4.9281 | <0.0001 | <0.0001 | 5479 |
| | EMTAB942_CAPRONI_COMMON_UP | 31 | 0.6080 | 4.0269 | <0.0001 | <0.0001 | 2302 |
| | EMTAB942_CAPRONI_ALUM_UP | 87 | 0.2310 | 2.5195 | 0.0002 | 0.0006 | 10393 |
| | GSE13485_QUEREC_D3_YF17D_UP | 113 | 0.1647 | 2.0513 | 0.0036 | 0.0095 | 7171 |
| | GSE13485_QUEREC_D3_YF17D_DOWN | 24 | 0.3194 | 1.8823 | 0.0108 | 0.0237 | 6642 |
| | EMTAB942_CAPRONI_ALUM_DOWN | 212 | 0.0976 | 1.6528 | 0.0318 | 0.0716 | 13000 |
| | GSE52245_LI_MPSV_UP | 136 | 0.1171 | 1.5891 | 0.0477 | 0.0892 | 3285 |
| | GSE13485_QUEREC_D1_YF17D_UP | 145 | 0.1118 | 1.5821 | 0.0470 | 0.0857 | 3991 |
| | GSE52245_LI_MCV_UP | 36 | 0.2131 | 1.5072 | 0.0744 | 0.1122 | 9166 |
| | GSE29615_NAKAYA_D3_LAIV_UP | 300 | 0.0730 | 1.4545 | 0.0860 | 0.1323 | 4689 |
| | ETABM506_MOSCA_CPG_COMBINED_DOWN | 28 | 0.2276 | 1.4347 | 0.0977 | 0.1355 | 2824 |
| | GSE13485_QUEREC_D1_YF17D_DOWN | 40 | 0.1344 | 1.0043 | 0.4246 | 0.5989 | 6241 |
| | ETABM506_MOSCA_CPG_COMBINED_UP | 82 | 0.0933 | 0.9937 | 0.4462 | 0.5852 | 11314 |
| | ETABM506_MOSCA_COMMON_DOWN | 10 | 0.2552 | 0.9895 | 0.4526 | 0.5623 | 7590 |
| | ETABM506_MOSCA_MF59_COMBINED_UP | 263 | 0.0464 | 0.8651 | 0.6196 | 0.7387 | 13749 |
| | ETABM506_MOSCA_ALUM_COMBINED_DOWN | 103 | 0.0705 | 0.8394 | 0.6560 | 0.7486 | 11060 |
| | ETABM506_MOSCA_ALUM_COMBINED_UP | 210 | 0.0375 | 0.6316 | 0.9170 | 0.9989 | 12689 |
| | EMTAB942_CAPRONI_MF59_DOWN | 187 | 0.0369 | 0.5878 | 0.9565 | 0.9922 | 4120 |
| | ETABM506_MOSCA_MF59_COMBINED_DOWN | 24 | 0.0880 | 0.5121 | 0.9876 | 0.9869 | 2054 |

| | Vaccination gene sets | Size | Enrichment Score | Normalized Enrichment Score | Nominal P-value | FDR q-value | Rank at Max |
|---|---|---|---|---|---|---|---|
| Nanopatch 3500 J/m$^2$ + vaccine + QS-21 | GSE89292_KAZMIN_ARR_DOWN | 346 | -0.1567 | -3.3491 | <0.0001 | <0.0001 | 4729 |
| | GSE89292_KAZMIN_RRR_DOWN | 345 | -0.0913 | -1.9561 | 0.0048 | 0.0306 | 4705 |
| | GSE29615_NAKAYA_D3_LAIV_UP | 300 | -0.0785 | -1.5793 | 0.0449 | 0.1475 | 4656 |
| | GSE52245_LI_MCV_DOWN | 53 | -0.1627 | -1.4118 | 0.1029 | 0.2310 | 5281 |
| | GSE29617_NAKAYA_D3_TIV_UP | 173 | -0.0783 | -1.1953 | 0.2333 | 0.4222 | 8835 |
| | GSE13485_QUEREC_D3_YF17D_UP | 113 | -0.0950 | -1.1721 | 0.2526 | 0.3809 | 9110 |
| | ETABM506_MOSCA_ALUM_COMBINED_DOWN | 103 | -0.0828 | -0.9884 | 0.4510 | 0.5813 | 2572 |
| | GSE52245_LI_MPSV_DOWN | 91 | -0.0676 | -0.7544 | 0.7718 | 0.8739 | 3165 |
| | GSE13485_QUEREC_D1_YF17D_DOWN | 40 | -0.0737 | -0.5513 | 0.9737 | 0.9719 | 1732 |
| | GSE29615_NAKAYA_D3_LAIV_DOWN | 348 | 0.4262 | 9.1548 | <0.0001 | <0.0001 | 3809 |
| | EMTAB942_CAPRONI_MF59_UP | 272 | 0.3376 | 6.4080 | <0.0001 | <0.0001 | 2109 |
| | GSE29617_NAKAYA_D3_TIV_DOWN | 231 | 0.3627 | 6.3607 | <0.0001 | <0.0001 | 4456 |
| | GSE89292_KAZMIN_RRR_UP | 335 | 0.2485 | 5.2643 | <0.0001 | <0.0001 | 7301 |
| | EMTAB942_CAPRONI_COMMON_UP | 31 | 0.6104 | 4.0193 | <0.0001 | <0.0001 | 3362 |
| | EMTAB942_CAPRONI_ALUM_DOWN | 212 | 0.2312 | 3.8727 | <0.0001 | <0.0001 | 6163 |
| | EMTAB942_CAPRONI_CPG_UP | 155 | 0.2362 | 3.4320 | <0.0001 | <0.0001 | 5691 |
| | GSE89292_KAZMIN_ARR_UP | 332 | 0.1560 | 3.2839 | <0.0001 | <0.0001 | 2580 |
| | ETABM506_MOSCA_COMMON_UP | 123 | 0.2504 | 3.2330 | <0.0001 | <0.0001 | 5467 |
| | GSE13485_QUEREC_D3_YF17D_DOWN | 24 | 0.4160 | 2.4349 | 0.0004 | 0.0008 | 3575 |
| | EMTAB942_CAPRONI_MF59_DOWN | 187 | 0.1507 | 2.4230 | 0.0006 | 0.0008 | 8404 |
| | EMTAB942_CAPRONI_ALUM_UP | 87 | 0.2125 | 2.3289 | 0.0010 | 0.0014 | 1883 |
| | GSE52245_LI_MPSV_UP | 136 | 0.1691 | 2.3011 | 0.0016 | 0.0015 | 5040 |
| | ETABM506_MOSCA_CPG_COMBINED_UP | 82 | 0.1665 | 1.7546 | 0.0223 | 0.0303 | 8198 |
| | GSE52245_LI_MCV_UP | 36 | 0.2071 | 1.4775 | 0.0788 | 0.1066 | 4055 |
| | GSE13485_QUEREC_D1_YF17D_UP | 145 | 0.1050 | 1.4692 | 0.0794 | 0.1038 | 8811 |
| | ETABM506_MOSCA_MF59_COMBINED_DOWN | 24 | 0.2402 | 1.4209 | 0.0963 | 0.1204 | 7990 |
| | ETABM506_MOSCA_MF59_COMBINED_UP | 263 | 0.0708 | 1.3292 | 0.1468 | 0.1664 | 5620 |
| | ETABM506_MOSCA_COMMON_DOWN | 10 | 0.3290 | 1.2720 | 0.1824 | 0.1965 | 6332 |
| | ETABM506_MOSCA_ALUM_COMBINED_UP | 210 | 0.0720 | 1.2118 | 0.2200 | 0.2325 | 4554 |
| | ETABM506_MOSCA_CPG_COMBINED_DOWN | 28 | 0.1759 | 1.1124 | 0.3052 | 0.3098 | 4924 |

| | Vaccination gene sets | Size | Enrichment Score | Normalized Enrichment Score | Nominal P-value | FDR q-value | Rank at Max |
|---|---|---|---|---|---|---|---|
| ID 50 J/m$^2$ + vaccine | GSE89292_KAZMIN_ARR_DOWN | 346 | -0.1161 | -2.5056 | <0.0001 | 0.0013 | 6836 |
| | GSE89292_KAZMIN_RRR_DOWN | 345 | -0.1044 | -2.2317 | 0.0016 | 0.0075 | 7403 |
| | GSE13485_QUEREC_D3_YF17D_UP | 113 | -0.1378 | -1.7119 | 0.0247 | 0.1186 | 11403 |
| | EMTAB942_CAPRONI_MF59_DOWN | 187 | -0.0954 | -1.5154 | 0.0603 | 0.2262 | 13079 |
| | GSE52245_LI_MPSV_DOWN | 91 | -0.1223 | -1.3661 | 0.1293 | 0.3418 | 11423 |
| | EMTAB942_CAPRONI_ALUM_UP | 87 | -0.1223 | -1.3324 | 0.1423 | 0.3258 | 1062 |
| | GSE89292_KAZMIN_ARR_UP | 332 | -0.0633 | -1.3270 | 0.1442 | 0.2851 | 5314 |
| | GSE13485_QUEREC_D1_YF17D_DOWN | 40 | -0.1644 | -1.2305 | 0.2092 | 0.3605 | 7012 |
| | ETABM506_MOSCA_CPG_COMBINED_DOWN | 28 | -0.1930 | -1.2193 | 0.2181 | 0.3337 | 7071 |
| | ETABM506_MOSCA_COMMON_DOWN | 10 | -0.2721 | -1.0516 | 0.3836 | 0.5266 | 5597 |
| | EMTAB942_CAPRONI_CPG_UP | 155 | -0.0728 | -1.0515 | 0.3711 | 0.4790 | 9006 |
| | GSE29615_NAKAYA_D3_LAIV_UP | 300 | -0.0506 | -1.0035 | 0.4372 | 0.5066 | 4897 |
| | GSE52245_LI_MCV_DOWN | 53 | -0.0941 | -0.8079 | 0.7096 | 0.7582 | 9988 |
| | GSE29617_NAKAYA_D3_TIV_UP | 173 | -0.0464 | -0.7169 | 0.8271 | 0.8265 | 1682 |
| | GSE29615_NAKAYA_D3_LAIV_DOWN | 348 | 0.1932 | 4.1265 | <0.0001 | <0.0001 | 3535 |
| | GSE29617_NAKAYA_D3_TIV_DOWN | 231 | 0.1982 | 3.4779 | <0.0001 | <0.0001 | 5747 |
| | EMTAB942_CAPRONI_MF59_UP | 272 | 0.1176 | 2.2581 | 0.0012 | 0.0052 | 7369 |
| | EMTAB942_CAPRONI_COMMON_UP | 31 | 0.2428 | 1.6053 | 0.0415 | 0.1707 | 818 |
| | ETABM506_MOSCA_MF59_COMBINED_UP | 263 | 0.0795 | 1.4924 | 0.0709 | 0.2315 | 9106 |
| | ETABM506_MOSCA_CPG_COMBINED_UP | 82 | 0.1293 | 1.3799 | 0.1204 | 0.3104 | 13614 |
| | GSE89292_KAZMIN_RRR_UP | 335 | 0.0645 | 1.3566 | 0.1257 | 0.2921 | 2128 |
| | EMTAB942_CAPRONI_ALUM_DOWN | 212 | 0.0782 | 1.3194 | 0.1531 | 0.2964 | 10027 |
| | ETABM506_MOSCA_MF59_COMBINED_DOWN | 24 | 0.1780 | 1.0382 | 0.3966 | 0.6969 | 11894 |
| | GSE13485_QUEREC_D3_YF17D_DOWN | 24 | 0.1639 | 0.9600 | 0.4879 | 0.7842 | 2893 |
| | ETABM506_MOSCA_COMMON_UP | 123 | 0.0710 | 0.9194 | 0.5437 | 0.7916 | 1015 |
| | GSE52245_LI_MPSV_UP | 136 | 0.0671 | 0.9155 | 0.5468 | 0.7324 | 3505 |
| | GSE52245_LI_MCV_UP | 36 | 0.1188 | 0.8470 | 0.6444 | 0.7967 | 4137 |
| | ETABM506_MOSCA_ALUM_COMBINED_UP | 210 | 0.0445 | 0.7492 | 0.7954 | 0.8960 | 10540 |
| | GSE13485_QUEREC_D1_YF17D_UP | 145 | 0.0470 | 0.6576 | 0.8987 | 0.9537 | 2028 |
| | ETABM506_MOSCA_ALUM_COMBINED_DOWN | 103 | 0.0533 | 0.6337 | 0.9178 | 0.9172 | 11682 |

**Supplementary Table 4**: Cell death information extracted from previous studies for the input of weighed gene co-expression network analysis (WGCNA).

| Sample | ID 50 $J/m^2$ + blank | ID 50 $J/m^2$ + vaccine | Nanopatch 3500 $J/m^2$ + blank | Nanopatch 3500 $J/m^2$ + vaccine | Nanopatch 3500 $J/m^2$ + vaccine + QS-21 | Nanopatch 11200 $J/m^2$ + vaccine | Study |
|---|---|---|---|---|---|---|---|
| **Cell death in a $16mm^2$ area of vaccinated site (Mean%)** | 3.7 | 3.1 | 16.5 | - | - | - | Depelsenaire et al 2014[3] |
| | - | - | - | 17.2 | 22.5 | - | Ng et al 2016[4] |
| **Cell death in a $16mm^2$ area of vaccinated site (Mean%)** | - | - | - | - | - | 24.3 | Current study (Supplementary Figure 5) |
| **Cell death information (Mean) extracted for WGCNA** | 3.7 | 3.1 | 16.5 | 17.2 | 22.47 | 24.3 | - |

**Supplementary Table 5**: All pathways analysed for all colour modules from the weighed gene co-expression network analysis (WGCNA).

**Supplementary Data 1.** Pathway enrichment using the Consensus Pathway Database, showing all pathways for all colour modules. In Supplementary Data File

## Supplementary Method

### The Nanopatch and the quantification of delivered vaccine using $^{14}C$ assay

The photos of Nanopatch™ compared to the conventional needles of different gauges. The concept of delivering vaccines using the Nanopatch™ is distinct from that of the needle and

syringe and a vaccine delivery experiment using $^{14}C$ tracer was performed to accurately calculate the vaccine dose delivered into the skin. Briefly, the tracer was mixed into the vaccine formulation, coated onto the patch and delivered the same way as described earlier. The Nanopatch™ was removed and the surface of the skin was gently swabbed six times with a dPBS moistened swab. The mice were euthanised and ears were excised. The Nanopatch™, swabs and excised ears were placed into separate scintillation vials. To extract the radioactivity from the patch and swab, 1 ml of PBS was added and vortex thoroughly. The ears were digested with Solvable (Perkin Elmer) at 60°C overnight. All samples were subjected to vigorous vortexing before the addition of 10 ml of scintillant (Ultima Gold™, Perkin Elmer). Disintegrations per minute (DPM) were measured for 10 minutes or a lower than 0.05% error protocol in a PerkinElmer TriCarb 2810 TR liquid scintillation analyser with automated quench correction and colour compensation. The amount of vaccine delivered to the skin was determined by the radioactivity present in the ear sample as a percentage of the total radioactivity in the ear, swab and Nanopatch™.

## Visualisation of enriched pathways using ClueGO

The ClueGO cytoscape plug-in[14] was used to visualise the enrichment of non-redundant biological terms in the KEGG pathways database. The genes used were selected from the modules that displayed significant positive or negative correlations with cell death and respective treatment groups. Genes from each module was assigned to their individual clusters for the analysis. Cluster distinction for enriched pathways was identified based on the criteria that > 50% of enriching genes were contributed from a given cluster/module and these pathways are highlighted with the respective colours. Pathways that display mixed contributions were coloured grey. Default settings for the analysis (version 2.5.0) was used. The KEGG database used was last updated on 20/11/17.

## References for Supplementary Information

1. Fernando, G.J.P. et al. Nanopatch targeted delivery of both antigen and adjuvant to skin synergistically drives enhanced antibody responses. *J Control Release* **159**, 215-221 (2012).
2. Ng, H.-I., Fernando, G.J.P. & Kendall, M.A.F. Induction of potent CD8+ T cell responses through the delivery of subunit protein vaccines to skin antigen-presenting cells using densely packed microprojection arrays. *J Control Release* **162**, 477-484 (2012).
3. Depelsenaire, A.C.I. et al. Co-Localization of Cell Death with Antigen Deposition in Skin Enhances Vaccine Immunogenicity. *J Invest Dermatol* **134**, 2361-2370 (2014).
4. Ng, H.-I., Fernando, G.J.P., Depelsenaire, A.C.I. & Kendall, M.A.F. Potent response of QS-21 as a vaccine adjuvant in the skin when delivered with the Nanopatch, resulted in adjuvant dose sparing. *Sci Rep* **6**, 29368 (2016).
5. Caproni, E. et al. MF59 and Pam3CSK4 Boost Adaptive Responses to Influenza Subunit Vaccine through an IFN Type I-Independent Mechanism of Action. *J Immunol* **188**, 3088-3098 (2012).
6. Mosca, F. et al. Molecular and cellular signatures of human vaccine adjuvants. *Proc Natl Acad Sci* **105**, 10501-10506 (2008).
7. Kazmin, D. et al. Systems analysis of protective immune responses to RTS,S malaria vaccination in humans. *Proc Natl Acad Sci* (2017).
8. Nakaya, H.I. et al. Systems biology of vaccination for seasonal influenza in humans. *Nat Immunol* **12**, 786-795 (2011).
9. Li, S. et al. Molecular signatures of antibody responses derived from a systems biology study of five human vaccines. *Nat Immunol* **15**, 195-204 (2014).
10. Querec, T.D. et al. Systems biology approach predicts immunogenicity of the yellow fever vaccine in humans. *Nat Immunol* **10**, 116-125 (2009).
11. Pearton, M., Pirri, D., Kang, S.-M., Compans, R.W. & Birchall, J.C. Host Responses in Human Skin After Conventional Intradermal Injection or Microneedle Administration of Virus-Like-Particle Influenza Vaccine. *Adv Healthc Mater* **2**, 1401-1410 (2013).
12. Zeng, J. et al. Identification and analysis of house-keeping and tissue-specific genes based on RNA-seq data sets across 15 mouse tissues. *Gene* **576**, 560-570 (2016).
13. Sudmant, P.H., Alexis, M.S. & Burge, C.B. Meta-analysis of RNA-seq expression data across species, tissues and studies. *Genome Biol* **16**, 1-11 (2015).
14. Bindea, G. et al. ClueGO: a Cytoscape plug-in to decipher functionally grouped gene ontology and pathway annotation networks. *Bioinformatics* **25**, 1091-1093 (2009).
